# The Association Between the Bared-Teeth Display and Social Dominance in Captive Chimpanzees (*Pan troglodytes*)

**DOI:** 10.1007/s42761-022-00138-1

**Published:** 2022-10-06

**Authors:** Yena Kim, Jolinde M. R. Vlaeyen, Raphaela Heesen, Zanna Clay, Mariska E. Kret

**Affiliations:** 1 Institute of Psychology, Cognitive Psychology Unit, Leiden University, Leiden, The Netherlands; 2 Institute of Cognitive Science, Comparative BioCognition, University of Osnabrück, Osnabrück, Germany; 3 Department of Psychology, Durham University, Durham, UK

**Keywords:** Emotional expression, Bared-teeth display, Chimpanzee, Homology, Power asymmetry hypothesis

## Abstract

Humans use smiles — widely observed emotional expressions — in a variety of social situations, of which the meaning varies depending on social relationship and the context in which it is displayed. The homologue of the human smile in non-human primates — both due to morphological and functional similarities — is the bared-teeth display (BT). According to the power asymmetry hypothesis (PAH), species with strict linear dominance hierarchies are predicted to produce distinct communicative signals to avoid escalations of social conflicts. Hence, while the BT in a despotic species is predicted to be expressed from low- to high-ranking individuals, signaling submission, the BT in a tolerant species is predicted to be expressed in multiple contexts, regardless of rank. We tested this hypothesis in a group of 8 captive chimpanzees (*Pan troglodytes*), a species commonly characterized as rather despotic. An investigation of 11,774 dyadic social interactions revealed this chimpanzee group to have a linear dominance hierarchy, with moderate steepness. A Bayesian GLMM — used to test the effects of social contexts and rank relationships of dyads on the use of the BT display — indicated multi-contextual use of the BT which is contingent on the rank relationship. We also found that slight morphological and/or acoustic variants (i.e., silent bared-teeth and vocalized bared-teeth) of the BT display may have different communicative meanings. Our findings are in line with the prediction derived from the PAH for a moderately despotic species, and the view that the human smile originated from the primate BT display.

**Supplementary Information:**

The online version contains supplementary material available at 10.1007/s42761-022-00138-1.

Humans — and possibly other animals too — cannot imagine a day without emotional experiences. Emotions are central to human life, playing a crucial role in coordinating and maintaining social interactions (Downs & Smith, 2004; Kashdan et al., 2013). Emotions are communicated through various verbal, as well as non-verbal channels among which the face conveys the most salient information about an individual’s internal and motivational states (Crivelli & Fridlund, 2018; Ekman, 2006; Fridlund & Russell, 2006; Horstmann, 2003). Ever since Darwin (1872), the question whether facial expressions are by-products of internal emotional states — which are innate and involuntary (Ekman & Keltner, 1997; Tomasello, 2010) — or whether they have been selected for communicative purposes (Fridlund, 1991) has been a long-standing debate. The current study thus aims at addressing this in chimpanzees (*Pan troglodytes*), one of humans’ closest relatives.

Studies on prenatal and neonatal infants (Izard et al., 1980; Kurjak et al., 2005) and people born blind (Matsumoto & Willingham, 2009) have demonstrated that some of the facial expressions of emotions, such as smiles, exist early in life. These are expressed indistinguishably between infants and adults, and between congenitally and noncongenitally blind individuals, supporting the view that facial expressions are biologically wired and tightly linked with felt emotions (Ekman & Keltner, 1997). However, smiles can also be “posed” or voluntarily controlled (Rinn, 1984), and are produced in multiple contexts, sometimes being on the opposite ends of the valence spectrum (positive, rewarding or affiliative smiles; negative, nervous or submissive smiles; Martin et al., 2017 ; Rychlowska et al., 2017). Although there are slight morphological differences between positive and negative smiles, all variants have a core feature which involves the zygomaticus major muscle (the lip corner puller, action unit 12; hereafter AU12), and function to reduce aggression and increase affiliation (Martin et al., 2017; Rychlowska et al., 2017). The abovementioned examples indicate that some facial expressions in humans are linked to felt emotions, which have acquired a communicative value, and are thus under voluntary control to adapt to social environments they live in (Ekman & Friesen, 1969; Matsumoto, 1990 for a review).

Non-human primates, our close evolutionary cousins, have a very similar facial muscle (i.e., zygomaticus major muscle, AU12; Powell et al., 2018) which activates when expressing bared-teeth displays (hereafter BTs). Due to similarities in morphology — lips being retracted and the teeth being exposed (Rychlowska et al., 2017; Van Hooff, 1967) — and function — communicating approachability or appeasement (Waller & Dunbar, 2005) — with human smiles, the BT has been proposed to be the origin of the human smile (Van Hooff, 1972). A majority of primates, in fact, have quite complex facial musculature, involving movements of the mouth and lips, eyelids and eyebrows, forehead, and the ears (Diogo et al., 2009), allowing them to produce a wide range of expressive facial movements (Van Hooff, 1967; Waller & Micheletta, 2013). Moreover, a number of primate species seem to have voluntary control over their facial movements (Florkiewicz & Campbell, 2021; Hopkins et al., 2011; Reynolds Losin et al., 2008), and an understanding of how facial expressions can predict future behaviors (Waller et al., 2016, 2017). It is also apparent that certain primates can maintain facial expressions longer when facing another individual, regardless of the emotional state (Scheider et al., 2016). These studies suggest that facial expressions of nonhuman primates are not merely cues of emotion, but have been selected for their communicative value to others as well (Oña et al., 2019; Petersen et al., 2018; Waller et al., 2020)—see Kret et al. (2020) and Heesen et al. (2021) for a thorough review.

Preuschoft and van Hooff (1995) earlier found that the BT — specifically the silent BT (SBT) — conveys different communicative meanings across closely related macaque species. This is tightly linked to species’ social characteristics (hierarchical structure), and thus, they proposed the power asymmetry of motivational emancipation hypothesis (hereafter PAH). According to the PAH, it should be particularly important for primate species with strict dominance styles — such as the rhesus (*Macaca mulatta*) and pig-tailed macaque (*Macaca nemestrina*; Beisner & McCowan, 2013; De Waal & Luttrell, 1985; Flack & De Waal, 2007) — to produce a distinct signal that is easily recognized by conspecifics to decrease escalations of fights. As such, the SBT should be used in narrow contexts, signaling short-term submission and long-term subordination, from subordinates to dominants. In contrast, in species with a more relaxed dominance style, the same facial expression is used more flexibly in broader contexts, usually during more positive social interactions, as is the case in Tonkean macaques (*Macaca tonkeana*; Demaria & Thierry, 2001) and moor macaques (*Macaca maura*; Petit & Thierry, 1992; Thierry, 2000). Although the communicative meanings of the SBT seem to differ across species, it ultimately functions to reduce aggression and/or increase affinitive contact (Bout & Thierry, 2005; Flack & De Waal, 2007; Waller & Dunbar, 2005). Remarkably, whether the PAH is also found in other species of primates, such as great apes, is currently unclear. Therefore, this study aims to further our understanding of the BT with regard to the PAH in chimpanzees.

Although not as despotic as rhesus macaques, chimpanzees are usually considered a rather despotic species (Boesch, 2009; Murray et al., 2007; Nishida, 2011; Wrangham, 1986) and often resort to aggressive behaviors in situations where tensions are high (Wilson et al., 2014). They are characterized by a high degree of fission–fusion social dynamics, and typically males dominate over females, with male dominance playing a crucial role in reproductive success (Wrangham, 1986; Wroblewski et al., 2009). The BT in chimpanzees has been described in a number of studies which report its usage in multiple social contexts, ranging from submission to affiliation to sexual interaction (Parr et al., 2005; Preuschoft & van Hooff, 1997; Van Hooff, 1967, 1972, 1973; Waller & Dunbar, 2005), communicating the signaler’s wishes of no harm or benign intent. Thus, it ultimately functions to reduce the risk of aggression and increase affinity (Van Hooff, 1967; Waller & Dunbar, 2005). Although a couple of studies have reported the use of BT by both dominant and subordinate individuals (Van Hooff, 1967, 1973), and by individuals within the same age-class (Waller & Dunbar, 2005), no systematic investigations of the BT in chimpanzees have been made under the PAH framework.

## Aim

The general aim of this study was to investigate the use of the bared-teeth display in a group of captive chimpanzees, to understand how the PAH hypothesis operates in a great ape species. More specifically, we investigated in which social contexts the BT was more likely to be produced, compared to the baseline (neutral context) and whether the BT was influenced by dyadic rank relationships. We further explored the difference between the silent bared-teeth (SBT) and vocalized bared-teeth (VBT) displays, as the context in which both displays are produced is known to differ (Van Hooff, 1967, 1973). We first investigated the dominance style of this group of chimpanzees, as dominance style of chimpanzees is known to vary across populations (Jaeggi et al., 2010; Kaburu & Newton-Fisher, 2015). Additionally, we explored whether the BT was influenced by social tension, independent of accompanying behaviors, to gain a better understanding of the communicative role of the BT in tension regulation.

## Method

### Study Subjects and Site

This study was conducted at ARTIS Amsterdam Royal Zoo, Amsterdam, the Netherlands. One group of chimpanzees, consisting of 8 individuals (1 sub-adult male, 2 adult males — one alpha, one castrate — and 5 adult females; Table S1) were housed in an enclosure with indoor/outdoor access all year. All individuals in the group had full contact with each other and were never separated. Chimpanzees had voluntary access to an indoor (261.5m^2^) and outdoor enclosure (206.6m^2^) all day and night, unless it was not possible due to cleaning or harsh weather conditions (e.g., stormy weather) to be outside. Observations were carried out in the indoor enclosure only, which is made of glass walls, to ensure maximum visibility of facial expressions, and due to practicability of video recordings. Due to the cold weather during the study period, the chimpanzees mostly stayed inside. The chimpanzees were fed regularly three times a day (in the early morning, at noon, and in the late afternoon). Additional food and non-food enrichment items, such as snacks, blankets, and cages with food, were provided throughout the day and water was available ad libitum.

### Data Collection

This study was a replication of the study by Vlaeyen et al. (in revision), which focused on bonobos, and thus, a similar methodology was used, adapted to the different physical environment. Data collection took place between March and May 2021. Observations were carried out by JMRV four days a week, from 10:30 to 17:45, unless there were slight changes of the caretakers’ cleaning/feeding schedule, in which cases we flexibly adjusted our observation schedule, ensuring that the overall data collected was balanced. Breaks were taken when the chimpanzees were locked outside for cleaning. All-occurrence sampling (Altmann, 1974; Martin & Bateson, 1993) was chosen over focal animal sampling to ensure sufficient number of facial expressions was collected for data analyses. Two cameras were used to record facial expressions and behaviors of the group. A hand-held video camera (SONY HDR-CX560V) was used specifically to zoom in upon social interactions between individuals to ensure the visibility of facial expressions, defined as when 2 or more individuals approached within 3 m, given limited space in the enclosure (Graham et al., 2018). A second camera, a Logitech BRIO Webcam, was placed stationary on the top part of the enclosure using a tripod, to record general behaviors of the group (see Figure S1), as well as any other behaviors that would be missed otherwise. Each video recording lasted approximately 20 min to facilitate coding afterwards, and additional information not visible on the recordings was spoken into the camera. When the social interaction of a target dyad stopped, we switched to another dyad engaging in a social interaction, while making sure the number of observed dyads was balanced. Social interactions of interest were affiliative, aggressive, neutral, sexual, submissive, and social play behaviors (for a detailed description, see Table 1 and Table S2).

**Table 1:** Grouped behavioral contexts and environmental conditions

Social contexts	
Affiliative	Affiliative touch; buddy walk; embrace; follow; give food; give; share food; grooming; hold genitals (also scrotum); hold hand; interfere; support; kiss; offer arm; reach hand; finger/hand in mouth; head nod; peering; sit together
Sexual	Mount; copulate; present; lead; leap bipedal on the spot; dart; inspect genitals; press teeth against back; sociosexual behavior
Social play	Play in rough and tumble; play socially with object; play walk; play-bite; rough play; tag; tickle; roll; play push; play slap
Aggressive	Aggression with attack; charge/chase; club; directed display; displacement; retaliate; shield; steal/take; tease; threat; throw at.
Submissive	Arm present*; avoid/yield; beg; bend away; bent wrist*; distress; flee; retreat; roll; rump present; submissive approach
Neutral	Approach; glance; move away; neutral contact; pass by
Environmental conditions	
Neutral	No-feeding.
Anticipation	Caretaker presence; anticipation for: feeding, changing enclosure.
Feeding	Feeding, feeding hand-given, feeding hand-given through door; feeding and caretaker presence.
Enclosure swap (non-feeding)	Changing enclosure without feeding; changing enclosure and caretaker presence without feeding.
Enclosure swap (feeding)	Changing enclosure with feeding; changing enclosure and caretaker presence with feeding.

References: (Cronin et al., 2015; Goodall, 1986; Hobaiter & Byrne, 2011; Nishida et al., 1999; Palagi, 2008; Parr et al., 2005; Pollick & De Waal, 2007; Van Hooff, 1973)

Difference between anticipation for food and caretaker presence is that caretaker presence meant no food involved after they had left again (e.g., busy in the kitchen without resulting in the chimpanzees getting food). *Depending on the context beforehand

In total, ±108 h of video material were recorded. Although we had some difficulties hearing soft vocalizations due to the glass windows between the chimpanzee and visitor area, all loud screams were audible. Further, due to the structure of the enclosure, some blind spots were available for the chimpanzees to hide from the camera, and thus, it was impossible to record all interactions. As such, it is important to note that mild vocalizations that accompany silent-bared teeth displays might have been misclassified.

#### Behavior Sampling/Coding Procedure

Two sources of video recordings of each observation were first synced in the program PluralEyes (PluralEyes, 2020). This was then imported into BORIS (Behavioral Observation Research Interactive Software; Friard & Gamba 2016), through which synced videos were analyzed together, following the ethogram created based on previously established studies (Cronin et al., 2015; Goodall, 1986; Hobaiter & Byrne, 2011; Nishida et al., 1999; Palagi, 2008; Parr et al., 2005; Pollick & De Waal, 2007; Van Hooff, 1973) and modified for the purpose of this study (Table S2).

Every social interaction with one or more recipients was coded as an event, indicating the initiator and recipient, in the same way as Vlaeyen et al. (in revision) did. If the recipient was unclear, we coded the recipient as “unspecified.” Importantly, for every interaction, the presence or absence of the BT was coded. Given that a social event often occurs in a series of behavioral exchanges among involved individuals which may convey different information (e.g., an aggressive interaction can be initiated by individual A aggressing individual B, which results in individual B displaying a submissive behavior to individual A), we coded every behavioral change produced by each individual as a discrete event. Therefore, in this case, individual A becomes the initiator of an aggression toward individual B (recipient) in the first event, and individual B becomes the initiator of a submission toward individual A (recipient) in the following event. If individual B displayed a facial expression in response to individual A’s aggression without any accompanying behavior, we coded only one event of individual A aggressing individual B with individual B’s facial expression in the comments (Figure S2). Further, when the face of the chimpanzees was partially or not visible, or if the quality of the video recording was not good enough to be certain of the facial expression, such events were coded as “out of sight” (16% of the analyzed data). When a behavior was ambiguous, it was coded as “other” and was excluded from further analysis. Additionally, due to the behavior arm present has been classified in multiple contexts (affiliative vs submissive; e.g., Bard et al., 2014), we divided them into categories depending on the context that happened right before; e.g., if an agonistic interaction occurred before an arm present, it was coded as submissive, otherwise it was coded as affiliative. It should be noted that unlike other studies investing the consequence of the display (e.g., gestures) in the recipient’s behavior to infer meaning or function of the display (Byrne et al., 2017; Graham et al., 2018), we were mainly interested in the immediate context triggering the bared-teeth display of the signaler. However, future studies would benefit from accounting for both signaler and recipient’s perspectives to fully understand the communicative use of the BT display.

#### Facial Expression Coding

BTs were coded by following the definitions created by Parr et al. (2005), one of the most comprehensive descriptions of chimpanzee facial displays (Table 2; Parr et al., 2005). The facial displays of chimpanzees were later validated by the Chimpanzee Facial Action Coding System (ChimpFACS; Parr et al., 2007). In this study, we primarily focused on the bared-teeth displays which were described either by the combination of AU10 (upper lip raiser) + 12 (lip corner puller) + 25 (lips parted) or by the combination of AU10 + 12 + 25 + 16 (lower lip depressor). Although the ChimpFACS did not distinguish between the silent bared-teeth (SBT) and vocalized bared-teeth (VBT), we additionally coded the VBT based on the presence of vocalizations that accompanied the BT to further explore the difference in the use of SBT and VBT. All other BTs without audible vocalizations were categorized into the SBT.

**Table 2:** Differences between SBT and VBT

	Description
Silent bared-teeth display (SBT)	The mouth may be slightly open or closed, lips withdrawn and mouth corners retracted laterally, and the teeth fully exposed. Eyes may be open or squinted. The lack of vocalizations helps define this from the other bared-teeth expressions.
Vocalized bared-teeth display (VBT)	The mouth can be partially open, corners are retracted, lips withdrawn with varying degrees of lateral lip retraction, but teeth are fully exposed. When very intense, wrinkles around the cheeks appear as mouth corners are obliquely retracted. Vocalizations are loud and high-pitched screams that are often very hoarse, can be voiced on the inhalation, and can sound like “aich-aich” panting or “eech” squeaks. These are usually sustained for several seconds, but can also quickly spasmodic, turning into a sustained tantrum/distress episode. Not to be confused with the open scream mouth, where the mouth is wide open, with lips fully withdrawn, exposing the teeth completely, and vocalizations include loud harsh screaming like “aach – aach”.

*Definitions from Parr et al. (2005). Examples of pictures can be found in Parr et al. (2005)

#### Tension Conditions

Further, to test the association between the BT and social tension, we created 5 different categories of external environmental conditions: neutral, anticipation, feeding, enclosure swap (non-feeding), and enclosure swap (feeding; Table 1), based the degree of potential tension involved. These conditions had to be adjusted to fit different enclosure styles than Vlaeyen et al. (in revision), to keep it as comparable as possible (for definitions see Table S3).

### Statistical Analyses

We recorded 14,962 events in total. We excluded events with multiple/unspecified recipients (*N*=1,527) and ambiguous behaviors (*N*=133) from the analysis. Additionally, as no BT was found during social play contexts, those events were not included in the analysis (*N*=1,528). As such, the final dataset consisted of 11,774 social events among which 9,808 observations have clear information on the presence and absence of the bared-teeth displays between 28 dyads (ranging from 7 to 22.7% across individuals). For the purpose of the study, only facial expressions of the initiator were analyzed.

#### Inter-rater Reliability

A randomly selected subset of the videos (*N*=20 videos including all individuals, amounting to 10% of all social interactions) was coded by a second coder (BvB), who was blind to the hypotheses. Detailed and comprehensive instructions were provided. BvB coded behavioral contexts, presence of BT, and recipient, already as grouped behaviors (Table 1). Inter-rater reliability was assessed by calculating Cohen’s Kappa and weighted Kappa, using the “kappa2” function in the irr package in R (Gamer et al., 2019). The agreement between the two coders was 0.846, which is considered excellent (Cicchetti, 2001; Cicchetti & Sparrow, 1981). Additionally, the reliability between the SBT and the VBT was also calculated, amounting to an agreement of 0.7, which is considered good (Cicchetti, 2001; Cicchetti & Sparrow, 1981).

#### Dominance Rank Analysis

For the social hierarchy rank analysis, we used dyadic agonistic interactions (both aggression and submission in response to aggression) of all individuals (*N* = 8) in the group who were socially and sexually mature (above the age of 7 years; Carlsen & de Jongh, 2007). Due to the window separating the observer and the chimpanzees, pant-grunts were not always heard and thus were not included in the analysis, reducing any potential bias. Based on the definitions by (Cronin et al., 2015; Goodall, 1986; Nishida et al., 1999; Parr et al., 2005; Table S2), the following aggressive behaviors were used: aggression with attack, charge/chase, club, direct display, displacement, retaliate, shielding, stealing/take, tease, threat, and throw at. Only submissive behaviors in response to aggression were used: bend away, flee, avoid/yielding, retreat, and roll. Two different matrices were created: one with all agonistic interactions, and one with all submissive interactions. To calculate the dominance hierarchy, the submissive matrix was transposed, and combined with the agonistic matrix (de Vries, 1995, 1998). First, the improved index of linearity (h_0_) was calculated with MatMan (de Vries et al., 1993) allowing for the possibility of tied and unknown relationships. To indicate a clear linear hierarchy, the index of linearity should be greater than 0.90 (de Vries, 1998). Additionally, to obtain a clear picture of dyadic dominance interactions, a complementary measure was calculated, namely the steepness of the hierarchy. The steepness measures the degree to which individuals differ from each other in dominant encounters, allowing for a difference in interactions between individuals (de Vries et al., 2006). The steeper the hierarchy, the more dominants win conflicts over subordinates. Using the “steeptest” function in the steepness package in R (Leiva & de Vries, 2014), we calculated the absolute slope fitted to normalized David’s scores, plotted against the subject’s ranks (de Vries et al., 2006). The steepness of dominance ranges from 0 — complete egalitarian society, or shallow hierarchy — to 1 — a steep or despotic hierarchy (de Vries et al., 2006; Van Schaik, 1989), and is dependent from the number of individuals.

#### Bared-Teeth Display Analyses

Given several benefits of Bayesian analyses over frequentist analyses, including robustness to model data with small sample sizes, allowance of prior knowledge in the model, and reducing type 1 errors (Hox et al., 2012; Van De Schoot et al., 2015), we fitted Bayesian generalized mixed models for all analyses, using the Stan computational framework (http://mc-stan.org/). Unlike the frequentist statistics, which give us the probability of observing the data under the null hypothesis, Bayesian statistics inform us about the reliability of the data of the parameters used, given the data observed (Kruschke et al., 2012; McElreath, 2018).

All models were fitted in R (version 4.1.2; R core Team, 2021) using the “brm” function in the *brms* package (Bürkner, 2017). All models included four MCMC (Markov Chain Monte Carlo) chains, with 4,000 iterations per chain. To ensure sampling calibration, 1,000 iterations were specified as warm-up, resulting in a total of 16,000 posterior samples. For all models, weakly informative priors on the intercept *α* ~ normal (0, 1), fixed effects *β* ~ normal (0, 1), and random effects *σ* ~ Cauchy (0, 1) were set to discourage overfitting and reduce inferential error (McElreath, 2018). For inference, we report multiple measures to summarize the posterior distributions for each parameter. In particular, to interpret the strength and uncertainty of estimated effects, we used the median estimate and the median absolute deviation (MAD), the 89% Bayesian credible interval (89% CI), and the probability of direction (*pd*), which varies between 50 and 100% and indicates the certainty with which an effect goes in a particular direction (Makowski et al., 2019). We checked model convergence by visually inspecting the trace plots, histograms of the posteriors and autocorrelation between iterations (Depaoli & Van de Schoot, 2017), and found no divergences with all R-hat statistics = 1.00, and no excessive autocorrelation (see Figure S3, S4, and S5 for details).

In the first model (model 1), we investigated the effect of the rank relationship of the dyad and social context on the use of bared-teeth display. The model was fitted to the Bernoulli response of the BT (binary coded as yes or no), with an interaction between the social context (five levels: affiliative, sexual, aggressive, submissive, neutral being the reference level) and relative dominance rank (two levels: to dominant, to subordinate being the reference level). We added sex (sum-to-zero coded to ease the interpretation of the intercept) of the initiator and recipient as fixed effects to control for variation in the use of bared-teeth display between males and females. Further, dyadic information (initiator, recipient, and their interaction) was included as random intercepts to account for individual as well as dyadic variation. We further explored whether the effect of the social context and rank differs between the silent bared-teeth displays and vocalized bared-teeth displays by running two separate analyses, one with the SBT displays (model 1a) and the other with the VBT displays (model 1b) as outcome variables (binary coded as yes or no). All other variables were the same as the BT model.

In model 2, we investigated whether the BT is associated with tension in the group while controlling for the rank and social context. The purpose of this analysis was to see whether chimpanzees use the BT more in high tension situations independent from the accompanying behavior and rank relationship, and therefore, the target variable of our interest was the environmental conditions associated with different levels of tension. This may tell us about the role of the BT with regards to tension regulation. Although previous studies have demonstrated that feeding can be a stressful factor in captivity (Paoli et al., 2007) and that chimpanzees use grooming and play behaviors to regulate tension in anticipation of feeding (Koyama & Dunbar, 1996; Palagi et al., 2004a), we verified this by checking whether feeding as well as other external environmental conditions (five levels: anticipation, neutral, feeding, enclosure swap [non-feeding], and enclosure swap [feeding]) actually increase the agonistic interactions in the group (model 2a). After confirming that feeding and anticipation of feeing conditions actually increases the probability of aggression (see Table S8), we fitted the model to the Bernoulli response of the BT (binary coded as yes or no). The second model was identical to the first model, except that we included an additional variable of the environmental condition (five levels: anticipation, neutral, feeding, enclosure swap [non-feeding], and enclosure swap [feeding]) as fixed effect.

## Results

### Dominance Hierarchy

Combining both aggressive (*N*=811, between all dyads (*N*=28)) and submissive behaviors (*N*= 1,002, between 26 dyads) resulted in a good measure for this group’s dominance hierarchy (significant linearity index *h*’=0.905, *P*=0.003). As the *h*’ index was above 0.9, the dominance hierarchy can be considered strictly linear (de Vries, 1998; Martin & Bateson, 1993). The steepness of this group was found to be moderate, with the slope being at 0.517 (*P*=0.005). The rank order derived from the analyses was assigned to the individuals (from high to low: Wakili > Margot > Leen > Vizuri > Amber > Ajani > Saphira > Quincy; see Table S4), and the relative rank relationship (to dominant vs. to subordinate) of each dyad was used for the subsequent analyses.

### Bared-Teeth Display

Of the analyzed data, the chimpanzees produced 337 bared-teeth displays during dyadic interactions (*N* = 11,774). All chimpanzees but the alpha male showed the bared-teeth at least once (Ajani 12.75% of the total BTs; Amber 2.67%; Leen 23.44%; Margo 19.88%; Quincy 2.67%; Saphira 36.20%; Vizuri 3.56%; Wakili 0%), in all social contexts but social play. The BT was also observed in every environmental condition, although only once during enclosure swap (non-feeding).

**Fig. 1:**
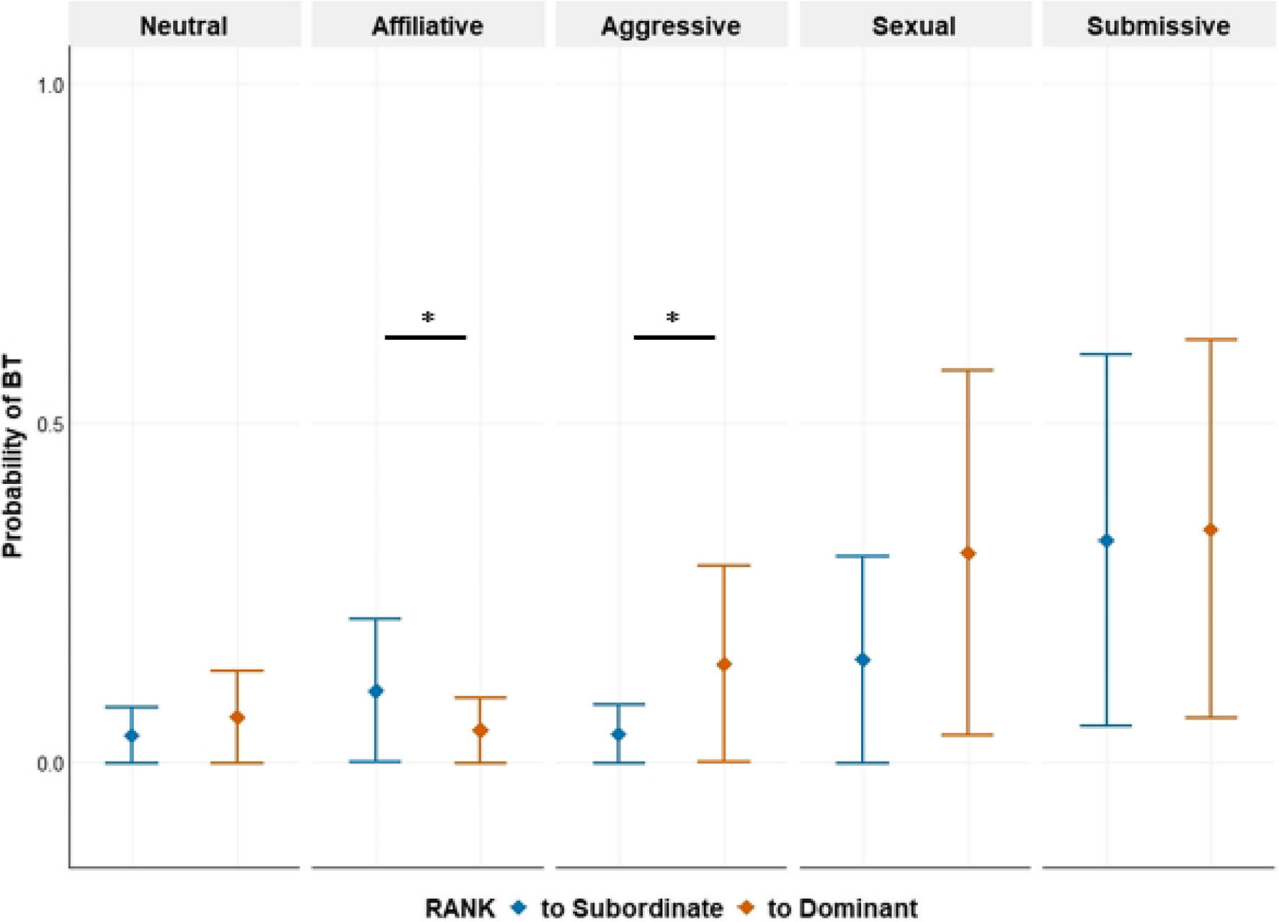
The predicted probability of bared-teeth displays in different social contexts and rank relationships. The upper and lower vertical lines represent standard errors and the diamonds represent the posterior median estimates. The interaction effect between the social context and rank revealed that the BT display was directed more toward subordinates than toward dominants in the affiliative social context, while the opposite was found in the aggressive social context. Lines with asterisks indicate robust differences (*pd*>0.97) between rank (to subordinate and to dominant)

The first model revealed a robust interaction effect between the social context and rank (Table 3; Fig. 1). To further investigate the interaction effect, we compared the likelihood of producing BT directed to dominants and subordinates for each behavioral context. The BT in the affiliative context were more directed toward subordinates, compared to dominants (median difference [MAD] = −0.07 [0.07], 89% CI [−0.2, −0.01], *pd* = 0.98), while the BT in the aggressive context were more directed toward dominants, compared to subordinates (median difference = 0.13 [0.12], 89% CI [0.03, 0.23], *pd* = 1.00). However, the BT directed toward dominants and subordinates did not differ in the other behavioral contexts (neutral: 0.03 [0.04], 89%CI [0, 0.12], *pd* = 0.93; sexual: 0.14 [0.12], 89%CI [0.02, 0.33], *pd* = 0.93, submissive: 0.01 [0.07], 89%CI [−0.08, 0.12], *pd* = 0.58). Importantly, the chimpanzees produced the BT more often in the submissive (median difference_submissive-neutral_ toward subordinates: 0.35 [0.2], 89%CI [0.13, 0.55], *pd* = 1.00; toward dominants: 0.33 [0.15], 89%CI [0.13, 0.46], *pd* = 1.00) and sexual contexts (median difference_sexsual-neutral_ toward subordinates: 0.14 [0.13], 89%CI [0.03, 0.36], *pd* = 1.00; toward dominants: 0.28 [0.17], 89%CI [0.1, 0.46], *pd* = 1.00), compared to the neutral context, regardless of the rank relationship. We further checked the possibility of the BT produced in the sexual context reflecting arousal or pleasure — the view proposed for bonobos (De Waal, 1988) — by manually checking the behaviors before and after the BT during sexual interactions in the videos (*N* = 14), and found out most sexual interactions (9 out of 14) where the BT was produced were non-copulatory behaviors and occurred after agonistic interactions.

**Table 3:** Posterior estimates for the fixed effects of the first model investigating the effect of social context and rank on the probability of BT

Fixed effect	Median estimate	MAD	89% CI lower bound	89% CI upper bound	*PD*
Rank (to dominant)	0.547	0.378	0.107	1.02	0.93
Social context (affiliative)	1.05	0.245	0.762	1.37	1.00
Social context (aggressive)	0.044	0.369	−0.399	0.488	0.55
Social context (sexual)	1.46	0.497	0.846	2.06	1.00
Social context (submissive)	2.47	0.293	2.12	2.83	1.00
Sex of the initiator (female)	0.183	0.762	−0.776	1.11	0.59
Sex of the recipient (female)	−0.482	0.151	−0.66	−0.29	1.00
Social context (affiliative): rank (to dominant)	−1.42	0.344	−1.84	−0.997	1.00
Social context (aggressive): rank (to dominant)	0.839	0.49	0.265	1.44	0.96
Social context (sexual): rank (to dominant)	0.383	0.581	−0.306	1.12	0.75
Social context (submissive): rank (to dominant)	−0.467	0.335	−0.869	−0.048	0.92

*Sex of the initiator and recipient was sum-to-zero coded. The parameters in bold indicate robust effects

Additional models for the silent bared-teeth (model 1a) and vocalized bared-teeth (model 1b) displays as separate outcome variables also found interaction effects between the social context and rank (see Tables S5 and S6). However, the pattern of the interactions was different. Whereas the effect of the rank on the probability of the SBT was found in the affiliative context (i.e., the SBT more directed toward subordinates than dominants; median difference [MAD] = −0.11 [0.11], 89%CI [−0.29, −0.02], *pd* = 0.98), but not in other contexts (neutral: 0 [0.04], 89%CI [−0.05, 0.08], *pd* = 0.56; aggressive: −0.02 [0.06], 89%CI [−0.16, 0.05], *pd* = 0.69, sexual: 0.13 [0.14], 89%CI [−0.01, 0.34], *pd* = 0.86, submissive: 0.1 [0.12], 89%CI [0, 0.3], *pd* = 0.88), the effect of the rank on the probability of the VBT was found in both the aggressive (i.e., VBT more directed toward dominants than subordinates; median difference [MAD] = 0.22 [0.17], 89%CI [0.06, 0.45], *pd* = 1.00) and affiliative contexts (i.e., VBT more directed toward subordinates than dominants; median difference [MAD] = −0.07 [0.07], 89%CI [−0.2, −0.01], *pd* = 0.95), but not in other contexts (neutral: 0.02 [0.03], 89%CI [−0.01, 0.11], *pd* = 0.82; sexual: 0.03 [0.09], 89%CI [−0.07, 0.21], *pd* = 0.66, submissive: 0 [0.08], 89%CI [-0.1, 0.12], *pd* = 0.52). Moreover, while the SBT was most pronounced in the sexual context, the VBT was most pronounced in the submissive context. Although it is interesting in itself, given the limited access to the vocalizations of the chimps, due to the glass windows between the chimpanzee and visitor areas, the results should be taken with caution.

### Bared-Teeth Display and Social Tension

Model 2 revealed that the chimpanzees were more likely to produce the BT when tension was presumably high, especially during the feeding condition (median estimate [MAD] = 0.542 [0.154], 89%CI [0.351, 0.733], *pd*=1.00), compared to the neutral condition (see Table S7 for details).

## Discussion

In this study, we investigated the use of the bared-teeth display in a group of captive chimpanzees, to understand how the PAH hypothesis operates in a great ape species. The main finding of the current study is that the BT is produced in multiple contexts, contingent on rank relationships. This resembles the communicative characteristics predicted for and found in species with tolerant dominance styles (Demaria & Thierry, 2001; Petit & Thierry, 1992; Preuschoft & van Hooff, 1995; Thierry, 2000). The dominance hierarchy in this group of captive chimpanzees, however, was found to be strictly linear, with moderate steepness, indicating modest power differences between adjacently ranked individuals. Given the previous assumption of despotic nature of chimpanzees (Boesch, 2009; Murray et al., 2007; Nishida, 2011; Wrangham, 1986) and linear hierarchy found in this group of chimpanzees, one might predict the BT to be used as a signal of submission by subordinates to dominants. As such, our findings do not seem to support the prediction derived from the PAH on the surface level. However, it should be noted that in primates dominance hierarchy or dominance style has been found to be a multifaceted continuum, ranging from egalitarian to despotic societies, depending on the aspects of aggressive interactions (e.g., intensity and directionality) and post-conflict behaviors (e.g., rate of reconciliation; Thierry, 2000). Given numerous previous findings on conflict resolution behaviors in chimpanzees (Koski et al., 2007; Palagi et al., 2004b; Watts, 2006) and a moderate steepness found in our group, this group of chimpanzees are clearly not as despotic as rhesus or Japanese macaques, but somewhere in the middle on the egalitarian-despotic spectrum, leaning more towards a despotic society. In that sense, the flexible and multi-contextual use of the BT in this group of chimpanzees seems to be in line with the PAH.

The flexible use of the BT also implies that it may not merely reflect internal states of the signaler, but serves communicative functions, flexibly adjusted depending on the context in which it is produced (Oña et al., 2019; Petersen et al., 2018; Waller et al., 2020). Specifically, the higher likelihood of BTs directed toward subordinates compared to dominants in the affiliative context is suggestive of reassurance signaling non-aggressive intent by dominants toward subordinates. On the other hand, the higher likelihood of BTs directed toward dominants compared to subordinates in the aggressive context may indicate subordinates’ motivation to appease dominants to reduce the probability of counterattack initiated by the dominant. Interestingly, the BT was as likely to be produced by both dominant and subordinate individuals in the submissive context, compared to the neutral context. Several alternative interpretations are possible; both dominants and subordinates have a similar communicative signal (i.e., formal signal of subordination) or internal state (i.e., fear associated with submissive behaviors), or they have different communicative signals. However, the BT in the submissive context does not suffice as a formal signal of subordination, acknowledging social status, as found in rhesus macaques (De Waal & Luttrell, 1985), as it was produced invariably between dominants and subordinates. It is more likely that the BT associated with submissive behaviors reflects an internal state of fear, especially if the BT was produced in response to aggression. Nonetheless, it is unlikely that the BT in general reflects an internal state of fear, as the BT produced by dominants in the affiliative context is less probable to be driven by fear. Another plausible explanation of the BT in the submissive context would be reassurance by dominants and appeasement by subordinates, as suggested by previous studies (Van Hooff, 1967, 1973).

Interestingly, we also found higher BTs by both dominants and subordinates in the sexual context, compared to the neutral context. A few studies have suggested that the BT reflects internal pleasure or arousal in bonobos (De Waal, 1988; Palagi et al., 2020) and chimpanzees (Nishida et al., 1999), none of which investigated it systematically however. Although we cannot eliminate the internal pleasure explanation, it seems very unlikely, as most sexual interactions in which the BT was produced were socio-sexual behaviors (9 out of 14) and occurred after agonistic interactions. Similarly, a recent study on same-sex sexual behaviors showed the victim to use a BT during the sexual behavior (Brooker et al., 2020). Thus, it seems that the BT during sexual behaviors signals appeasement and reassurance to regulate the tension elevated from the prior agonistic interactions. Similar results were found in bonobos, who also used the BT during socio-sexual behaviors, to reduce tension (Vlaeyen et al., in revision). The model investigating the association between the BT and external environmental conditions with varying degree of tension (model 2) supports this suggestion: In the feeding condition where social tension in the group was high — indicated by higher aggressive interactions — the BT was produced more likely than in the baseline neutral condition. It should be noted that the higher probability of the BT in the feeding condition is not the result of the higher aggressive interactions, as those behaviors were controlled for in the model.

Additional exploration of the difference between the use of silent and vocalized bared-teeth displays yielded interesting results, suggestive of potentially different communicative meanings. While the difference between the SBT directed toward dominants and subordinates was only found in the affiliative context, the difference between the VBT directed toward dominants and subordinates was found in both the affiliative and aggressive contexts. Moreover, unlike the SBT most pronounced in the sexual context, the VBT was most pronounced in the submissive context. van Hooff (1973) reported a similar pattern, where the SBT was associated with affinitive behaviors and the VBT with submissive behaviors. The morphological variance associated with different behavioral contexts potentially resembles the multi-contextual and social status dependent use of smiles in humans (Martin et al., 2017; Mehu & Dunbar, 2008). Such smiles with subtle morphological variance signals different meanings, ranging from affiliation to submission (Martin et al., 2017; Rychlowska et al., 2017). Additionally, crested macaques — the most socially tolerant species of macaques (Petit et al., 1997) — also have slight morphological variations, associated with different social outcomes: during submissive behaviors, the BT included teeth chatter and a high intensity of lip movements, whereas during copulation, a jaw wobble was present (Clark et al., 2020). Currently, it is still unclear whether the BT accompanying vocalizations was used to increase the salience of the signal or used as a multi-modal signal to deliver a different communicative meaning (Genty et al., 2014; Oña et al., 2019), as the current study did not use ChimpFACS to code BTs, and due to limited access to vocalizations accompanying BTs. Nonetheless, the finding that the VBT, but not the SBT, was most pronounced in the submissive context, suggests that morphological and/or acoustic variants of the BT in chimpanzees could potentially deliver different communicative meanings. Especially, the VBT with increased urgency would be more beneficial to avoid ambiguity, compared to the SBT, as miscommunication of submission could be harmful (Clark et al., 2020).

Taken together, our findings are in line with the prediction derived from the PAH in that chimpanzees, as a species with moderately despotic dominance style, use the BT in a wide range of contexts, of which the meaning is dependent on the context in which it is displayed, as well as the rank relationship. Furthermore, the BT is used as a communicative tool to regulate social tension. Future studies would benefit from applying ChimpFACS to coding facial expressions, as well as incorporating the behavioral consequence of the BT to further illustrate communicative meanings and functions of morphological variants of the BT. Moreover, comparative studies across closely related species, as well as populations with varying degree of dominance style within the same species should follow to better understand the evolution and ontogeny of the BT and the impact of social environment on the signal use and functionality. Finally, the BT display in combination of other communicative modalities, such as gestures and vocalizations should follow to fully comprehend the great ape communication (Genty et al., 2014; Oña et al., 2019).

## Supplementary Information

ESM 1 (DOCX 1.48 mb)

## Additional information

### Data Availability

The data that support the findings of this study are openly available in Figshare at 10.6084/m9.figshare.18480707.
