# Supplementary material for: The Association Between the Bared-Teeth Display and Social Dominance in Captive Chimpanzees (Pan troglodytes)
Source: Affect Sci. 2022 Oct 6;3(4):749–60. doi: 10.1007/s42761-022-00138-1 (PMC9535227; doi:10.1007/s42761-022-00138-1)
Supplement: Supplementary file 1 — (DOCX 1.48 mb) [file 42761_2022_138_MOESM1_ESM.docx]

**Supplementary materials for**

**The association between the bared-teeth display and social dominance in captive chimpanzees (*Pan troglodytes*)**

Yena Kim^1*^, Jolinde M.R. Vlaeyen^1,2*^, Raphaela Heesen^3^, Zanna Clay^3^, and Mariska E. Kret^1^

^1^ Institute of Psychology, Cognitive Psychology Unit, Leiden University, The Netherlands

^2^ Institute of Cognitive Science, Comparative BioCognition, University of Osnabrück
^3^ Department of Psychology, Durham University, UK

*Corresponding author:* Yena Kim, y.kim@sfw.leidenuniv.nl

* Joint first authorship

1. METHODS

Table S1. Individuals observed during this study (N=8). Individual in bold is the alpha male. Age classes followed according to Goodall (1986) and Carlsen and de Jongh (2007).

| **Individual** | **Sex** | **Age class** | **Age & (Year of birth)** | **Mother (in group)** |
| --- | --- | --- | --- | --- |
| Ajani | M | Sub-Adult | 8 (2013) | Amber |
| Amber | F | Adult | 27 (1994) | - |
| Leen | F | Adult | 40 (1981) | - |
| Margo | F | Adult | 52 (1969) | - |
| Quincy | F | Adult | 33 (1988) | - |
| Saphira | F | Adult | 20 (2001) | - |
| Vizuri | M | Adult | 23 (1998) | Leen |
| **Wakili** | M | Adult | 18 (2003) | Amber |


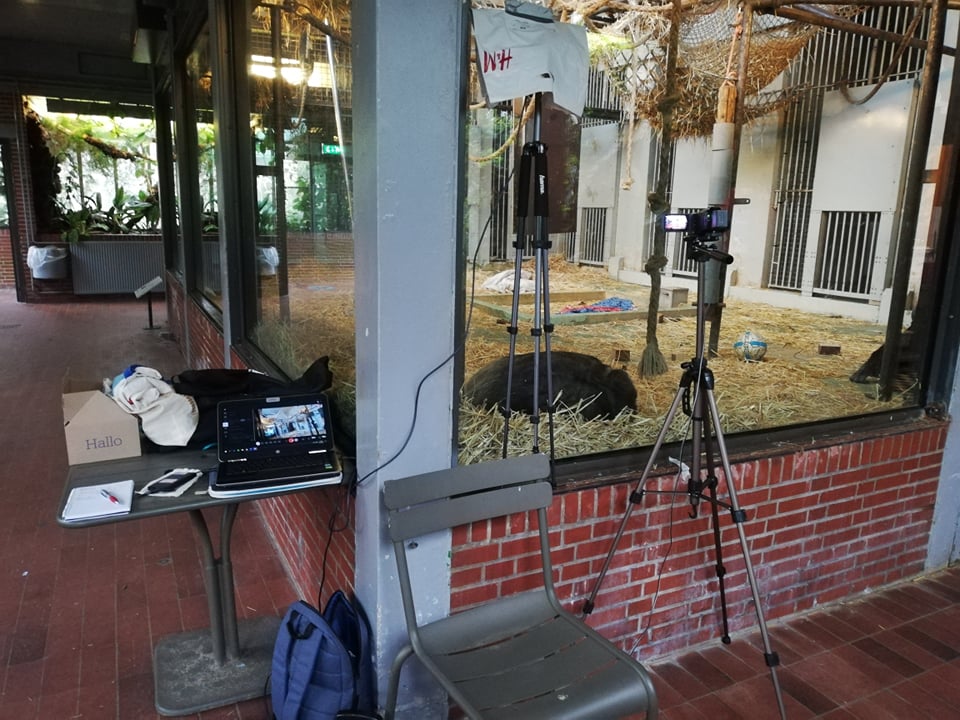


**A.**

**B.**

Figure S1. Setup of the 2 different cameras: the Logitech Brio Webcam (A) and the SONY camera (B). The webcam was placed stationary on a tripod on one side of the enclosure, to record general behaviors that occurred on the top part of the enclosure. The SONY camera was used specifically to zoom in upon social interactions between individuals.

Table S2. Ethogram created for this study, based on previously established studies (Cronin et al., 2015; Goodall, 1986; Hobaiter & Byrne, 2011; Nishida et al., 1999; Palagi, 2008; Parr et al., 2005; Pollick & De Waal, 2007; Van Hooff, 1973) and modified for the purpose of this study.

| **Behaviors** | **Definitions of behaviors** | **Source** |
| --- | --- | --- |
| **AFFILIATIVE** *- Includes all behaviors below* | | |
| Affiliative touch | Subject touches the body of the receiver with hand or fingers in a relaxed and non-aggressive manner. Also scored after a third party consoles a victim. | Cronin et al., (2015); Nishida et al. (1999) |
| Buddy walk | Subject walks next to receiver, has put an arm on the shoulder of receiver | Cronin et al., (2015) |
| Embrace | Gentle contact to another individual using the arms or another body part: subject gently places one arm around receiver's shoulder, back or waist, or putting both arms around the other while pulling the other closer. | Parr, Cohen & de Waal (2005); Cronin et al., (2015) |
| Follow | Walk after, trying to maintain close proximity (but not physical contact) to another individual. | Nishida et al. (1999) |
| Give Food | Hand over food by the owner to another who was begging. | Nishida et al. (1999) |
| Give | Hand over food by the owner to another was not begging. | Nishida et al. (1999) |
| Share food | Allow another individual to take food that is under the control of the owner (for example, in the mouth or hand, on the lap, or in proximity). | Nishida et al. (1999) |
| Grooming | Use both hands, pushing the hair back with the thumb or index finger of one hand and holding it back while picking at the exposed skin with the nail of the thumb or index finger of the other. | Nishida et al. (1999); Parr, Cohen & de Waal (2005); Goodall (1989) |
| Hold genitals (also scrotum) | Adult usually grabs or touches the scrotum or penis of a male as reassurance. Often occurs when an adult male is mounted by another male, the mounted reaches his hand between his thighs and holds the scrotum of the mounter. When an adult female pant-grunts to an adult male she sometimes holds his scrotum. | Nishida et al. (1999); Goodall (1989) |
| Hold hand | The most commonly seen form of holding. This occurs when a higher-ranking individual grasps the stretched out hand of a lower ranking chimp. | Nishida et al. (1999); Goodall (1989) |
| Interfere | A pattern of intervention (see Intervene) in which "a third individual prevents or tries to prevent an interaction between two others." | Nishida et al. (1999); Goodall (1989) |
| Support | One individual comes to the aid of another who is engaged in a dyadic conflict. The supporter does not have to be recruited. Intervene in a fight and side with either of the conflicting parties. | Parr, Cohen & de Waal (2005); Nishida et al. (1999) |
| Kiss | Mouth contact on the mouth, or body of another individual. If mutual, note for both individuals. Indicate if panting is seen/heard. | Parr, Cohen & de Waal (2005); Nishida et al. (1999) |
| Offer arm | Individual extends arm to another to allow the latter to mouth the arm to be reassured. | Nishida et al. (1999) |
| Reach hand | One individual holds out their hand to another with wrist positioned first and bent. This is often forced into the face of the recipient. | Parr, Cohen & de Waal (2005); Nishida et al. (1999) |
| Finger/hand in mouth* | Putting a finger or hand into another individual's mouth | Pollick & de Waal |
| Head nod | Repeated back and forth movement of the head | Hobaiter & Byrne., 2011 |
| Peering | Subject stares into the face of the receiver from a distance of less than 30cm for at least 5 sec. Look intently into another individual's face from a few cm. | Nishida et al. (1999) |
| Sit together | Sitting within one arm's length of another individual. Only coded when directed to the other individual. Not scored when scoring grooming. | Vlaeyen et al., (in revision) |
| **AGGRESSIVE –** *Coded separately* | | |
| Aggression with attack | Hitting, biting, trampling, leaping on, biting, or otherwise attacking another individual. | Parr, Cohen & de Waal (2005) |
| Charge/  chase | Subject shows tense running toward the receiver. Run after a fleeing individual, in order to grab it in aggression. | Cronin et al., (2015); Nishida et al. (1999); Van Hooff (1973); Goodall (1989) |
| Club | Strike target with a long (usually more than 1m), thick stick in overthrow movement. Club another individual with a stick. | Nishida et al. (1999); Van Hooff (1973); Kortlandt and Kooij (1963) |
| Direct display | Tense running in the direction of, or parallel to, or closely passing by another individual usually while pushing an object, resulting in collision or other contact aggression | Cronin et al., (2015) |
| Displacem-ent | Subject approaches receiver and forces receiver to leave. Drive off another chimpanzee from a food patch. | Cronin et al., (2015); Nishida et al. (1999) |
| Stealing/  Take | Take forcibly an object such as food from another individual without consent. Adult males snatch food, meat in particular, from individuals of any age-sex class, mothers from immature offspring, and juvenile sons from mothers. | Nishida et al. (1999) |
| Tease/ Pestering | Adolescent males pester and threaten adult females by <slap>, <hit>, and <throw branch>, <charge>, and so on until females pant-grunt to them. Adolescent or juveniles throw branch at another adolescent or juvenile apparently to tease him/her. | Nishida et al. (1999); Adang (1984) |
| Threat | Threatening behavior including arm waving and/or lunging. Response of the recipient is the best defining feature. Intention movement or preparatory gesture of aggression. Sudden tense hand/body movements in the direction of another individual in a non-playful context, or hitting or kicking another individual without locomotion | Parr, Cohen & de Waal (2005); Cronin et al., (2015); Goodall 1989 |
| Throw at | When an object is aimed at a specific objective. Aimed throwing. | Nishida et al. (1999); Goodall (1989) |
| Distress | A prolonged response by the recipient of aggression where victim persists in their negative response over 10 s. after initial incident. Negative response includes screaming, fleeing, looking for support, temper tantrums, rolling, etc. | Parr, Cohen & de Waal (2005) |
| Retaliate | Counter-attack, namely attack returned to the initial attacker. "Aggressive response directed toward the original aggressor by a chimp who has been threatened or attacked." | Nishida et al. (1999); Goodall (1989) |
| Shielding | Subject positions himself between receiver and a food item, object or infant apparently desired by receiver | Cronin et al., (2015) |
| **SUBMISSIVE –** *Coded separately* | | |
| Avoid/Yielding | When the approach of another individual leads the focal subject to move immediately away >1 m. Locomotion away from a partner. | Parr, Cohen & de Waal (2005); Nishida et al. (1999); |
| Beg (with hand or mouth) | Beg for food, toy, or any desirable object from the possessor. Begging is often accompanied by whimpering, and if unsuccessful, the beggar may even throw a tantrum. | Nishida et al. (1999); Goodall (1989) |
| Bend away | With elbow and wrist flexed, arm drawn close to body, the chimp leans slightly away from a passing higher-ranking animal. | Nishida et al. (1999); Goodall (1989) |
| Flee | When the approach of another individual leads to rapid avoiding by running, or climbing a structure. Pursuit may be the cause. Subject moves away quickly from receiver after receiver has behaved aggressively towards subject. | Parr, Cohen & de Waal (2005); Cronin et al., (2015); Nishida et al. (1999) |
| Submissive approach | Includes *bob* and *formal approach.* The body bobs up and down as elbows are flexed and straightened. Shown typically by adolescent males but also by adolescent females and juvenile and adult males when a high-ranking individual passes. Bending or crouching in front of a more dominant individual accompanied by pant-grunts (scored separately). | Nishida et al. (1999) |
| Retreat | Walk backwards on all fours. When a subordinate pant-grunts to a dominant, the subordinate occasionally walks backwards in fearful way. | Nishida et al. (1999) |
| **SEXUAL** *- Includes all behaviors below* | |  |
| Mount | Embrace another with both arms from behind. Part of the ventral surface of the mounter is in contact with part of the dorsal surface of the other, and the mounter leans forward over the other, usually grasping him/her. | Nishida et al. (1999); Goodall (1989) |
| Copulate | Intromission and pelvic thrusting between male and estrous female. | Nishida et al. (1999) |
| Present | Normal quadrupedal stance with hind quarters directed to another individual. The male sits with thighs splayed and penile erection, looking toward a female in estrus | Goodall (1989) |
| Lead | Mature male or female, and especially adolescent male, leads sexual partner into undergrowth or higher in a tree in order to avoid interference by more dominant males. | Nishida et al. (1999) |
| Leap bipedal on the spot | Juvenile male jumps on his feet in front of an estrous female in courtship, to elicit her to "present". | Nishida et al. (1999) |
| Dart | Estrous female runs quickly a few meters after copulation. Often accompanied by copulatory squeal and grin, then, the female often lies down for a few minutes. | Nishida et al. (1999) |
| Inspect genitals | When a male or female touches the vaginal opening of a female and sniffs finger, or sniffs with nose directly. Both hands may be used to pull apart the lips of vagina. | Nishida et al. (1999) |
| Press teeth against back | Adult male mounts another male, and the mounter often presses his upper row of teeth against the back of the mounted male, while the mounter embrace s the latter, performs a pelvic thrust and shows a wide open grin. | Nishida et al. (1999) |
| Sociosexual behavior | i.e. sexual behavior used in a nonreproductive context. | Parr, Cohen & de Waal (2005) |
| **SOCIAL PLAY***- Includes all behaviors below* | |  |
| Play in rough and tumble | Two or more youngsters grab hold of each other and often roll over as they bite, tickle, kick and so on without losing contact | Nishida et al. (1999) |
| Play socially with object | Social play including chasing an individual who has an object such as a fruit, branch, stone and animal skin and so on. | Nishida et al. (1999) |
| Play walk | The chimp walks with a rounded-back, its head slightly bent down and pulled back between the shoulders while it takes small stilted steps | Nishida et al. (1999) |
| Play-bite | Contact made on the partner's body with teeth. Sometimes one of the players gets a firm grip on a limb and holds on for minutes at a time. | Nishida et al. (1999) |
| Rough play | Play that includes restraining, or biting, or otherwise behavior that would be considered agonistic. This is also coded if the response of the partner is agonistic. | Parr, Cohen & de Waal (2005) |
| Tag | Play tag. Alternate chase and flee. | Nishida et al. (1999) |
| Tickle | One individual places one or both hands on the body of the partner, usually between the neck and shoulder or in the groin, and makes tickling movements with the fingers | Nishida et al. (1999) |
| Roll | A chimpanzee may hit another and roll over him during play | Nishida et al. (1999) |
| Play Push | An animal pushes a playmate with either its hands or its feet | Palagi, 2008 |
| Play Slap | An animal gently bites a playmate | Palagi, 2008 |
| **NEUTRAL -** *Includes all behaviors below* | |  |
| Approach | One individual approaches another within 2 m with no contact. This is only coded when the approach is observed. | Nishida et al. (1999) |
| Move Away | Move away from another individual. | Parr, Cohen & de Waal (2005) |
| Neutral behavior | Sitting, ignore others without contact or response. This is coded if a facial expression is made with no obvious accompanying behavior, or within a dyadic interaction as a neutral response category. | Nishida et al. (1999) |
| Neutral contact | Ambiguous contact by one individual towards another. The contact is not easily defined as play or aggression, i.e. touching. | Cronin et al., (2015); Nishida et al. (1999) |
| Pass by | Walks past another, with or without body contact. | Nishida et al. (1999) |
| **OTHER** - Any other behavior seen which is not in the ethogram - written in comments. | |  |


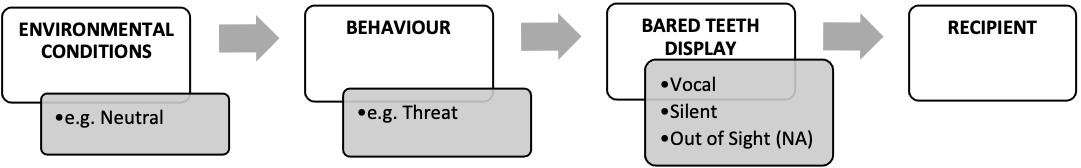


**INITIATOR**

Figure S2. Coding steps for the video recordings with BORIS software (Friard & Gamba, 2016), based on Vlaeyen et al. (in revision). As soon as a social interaction was visible, the condition in which it occurred was scored. Then, the social behavior of the initiator was scored, and written if a bared-teeth display was visible. Lastly, the recipient was also scored. If the recipient performed a facial expression without a behavior, it was written in the comments. If the recipient performed its own behavior, the recipient then became the initiator.

Table S3. *Environmental conditions based on de Waal (1988) and modified from (Vlaeyen et al., in revision). Any context could happen at the same time (e.g. Feeding time & Caretaker). Anticipation contexts were only scored based on the outcomes.*

| **Grouped conditions** | **Conditions** | **Definition** |
| --- | --- | --- |
| **Neutral** | *Non-feeding* | Any context that does not involve food. Includes eating branches and enrichment that is available all day long. In this condition, less tension is expected. |
| **Anticipation** | *For Feeding* **°** | Provision of browse food is at hand, as evident from the arrival of the animal caretakers and/or sounds in the kitchen. Scored from 10 minutes before they receive food from caretaker. In this condition, chimpanzees could see/hear the caretaker in the kitchen preparing food and actually receive food from the caretaker. |
|  | *Caretaker presence* | Caretaker walks into the kitchen, and is busy inside the kitchen. Difference between feeding anticipation if after caretaker presence they receive food before caretaker leaves again. In this condition, chimpanzees could see/hear the caretaker in the kitchen but the caretaker left the kitchen without having given food to the chimpanzees (e.g., medication preparation). |
|  | *For*  *Enclosure Swap* | Chimpanzees show restless behaviors due to the possibility of changing enclosures. Scored from 10 minutes before the enclosure swap actually happened (when the hatch to the outdoor enclosure opened). As we only filmed from inside, anticipation to inside enclosure never observed. |
| **Feeding** | *Feeding time***°** | Food is present in the form of vegetables or enrichments. Scored until the main food is gone. Does not count branches nor enrichments that are in the enclosure all day. |
|  | *Feeding time -  Hand Given* | Food is given by hand to the animals instead of being placed in the enclosure (through the mesh). Scored until the main food is gone. |
|  | *Feeding time -  Hand Given (Door)* | Food is given by hand to the animals instead of being placed in the enclosure (through the mesh door). Scored until the main food is gone. Mainly monopolizable items in this condition. |
| **Enclosure Swap** | *Enclosure Swap (Non-Feeding)* | Chimpanzees change enclosures and *no food* is present in the enclosure. Scored for 10 minutes after the enclosure swap happened (e.g. when the hatch to a new enclosure opened). |
|  | *Enclosure Swap (Feeding)* | Chimpanzees change enclosures and *food* is present in the enclosure. Scored for 10 minutes after the enclosure swap happened (e.g. when the hatch to a new enclosure opened). |
| ***Included in any of the above*** | *Response to inside***°** | Response to a disturbance inside their enclosures, behind the glass walls. |
|  | *Response to outside***°** | Response to the disturbance outside of the enclosure, such as when visitors are coming in, or when noises are the definite cause of behavior |
| *Sources:* Vlaeyen et al (in revision); **°**de Waal (1988). | | |


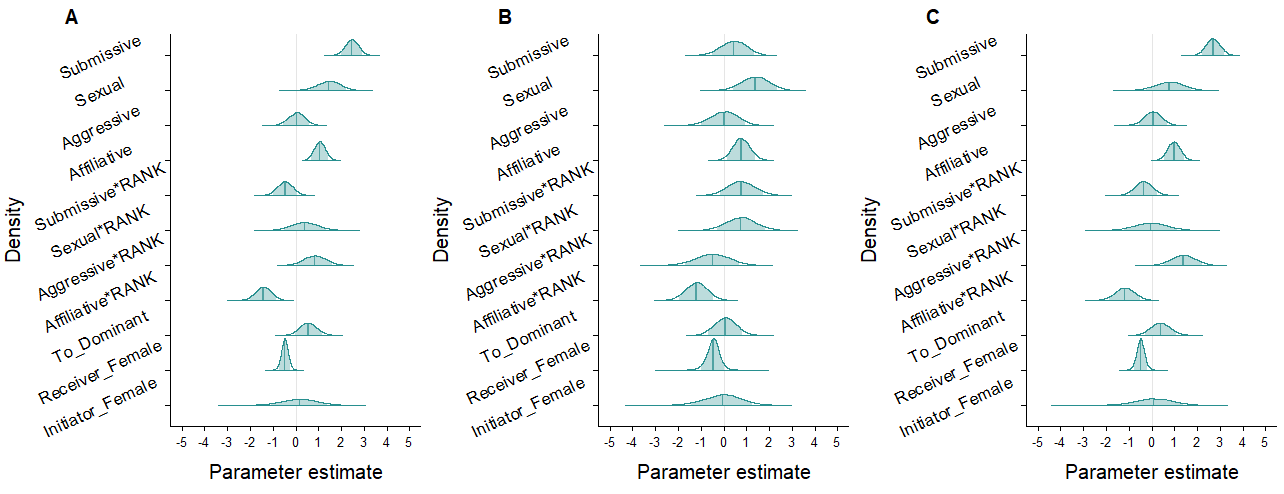


*Figure S3.* Posterior distributions and 89% Credible Intervals for each Bayesian generalized linear model, including all effects. **A****.** Density plot of model 1 representing the effects of rank and social contexts on the *bared-teeth display*. **B.** Density plot of model 1a representing the effects of rank and social contexts on the *silent* *bared-teeth display*. **C.** Density plot of model 1b, representing the effects of rank and social contexts on the *vocalized* *bared-teeth display*.

*
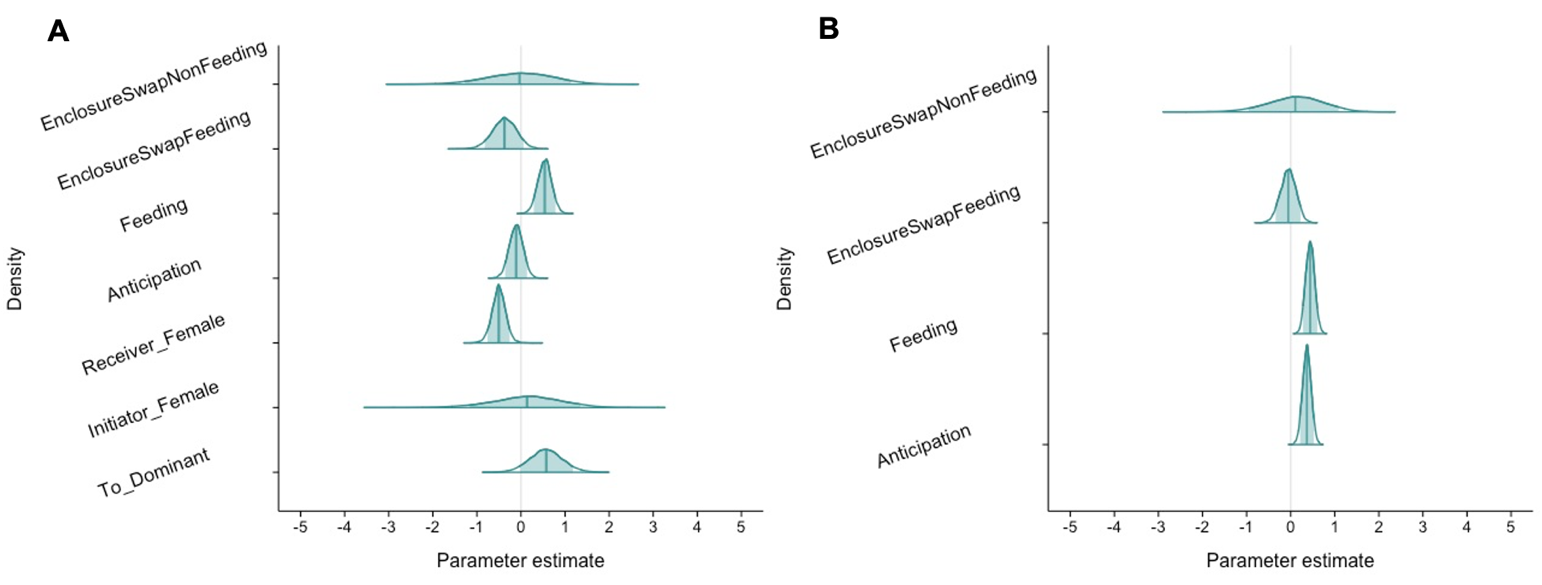
*

*Figure S4.* Posterior distributions and 89% Credible Intervals for each Bayesian generalized linear model, including all effects. **A.** Density plot of model 2 representing the effects of feeding conditions on the bared-teeth display. **B.** Density plot of the mode 2a, representing the effects of feeding conditions on aggressive behaviors.

**
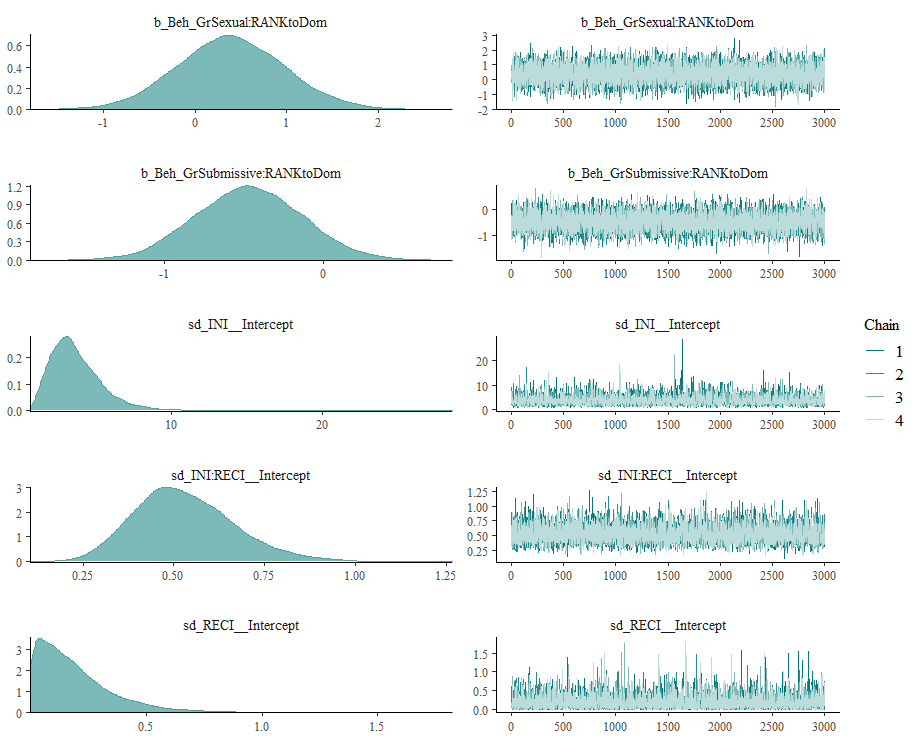

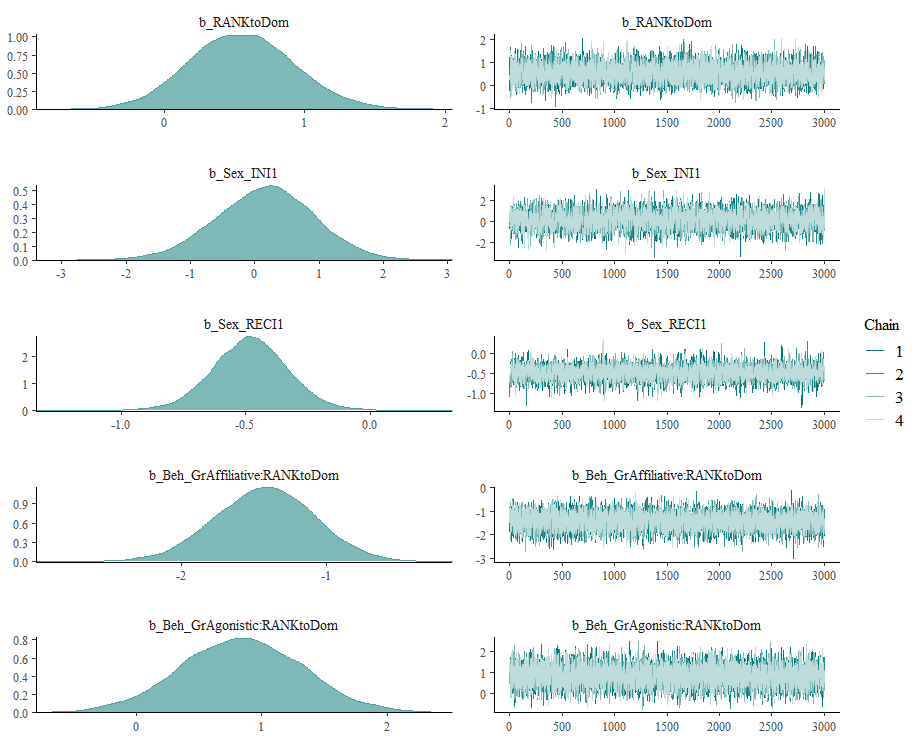

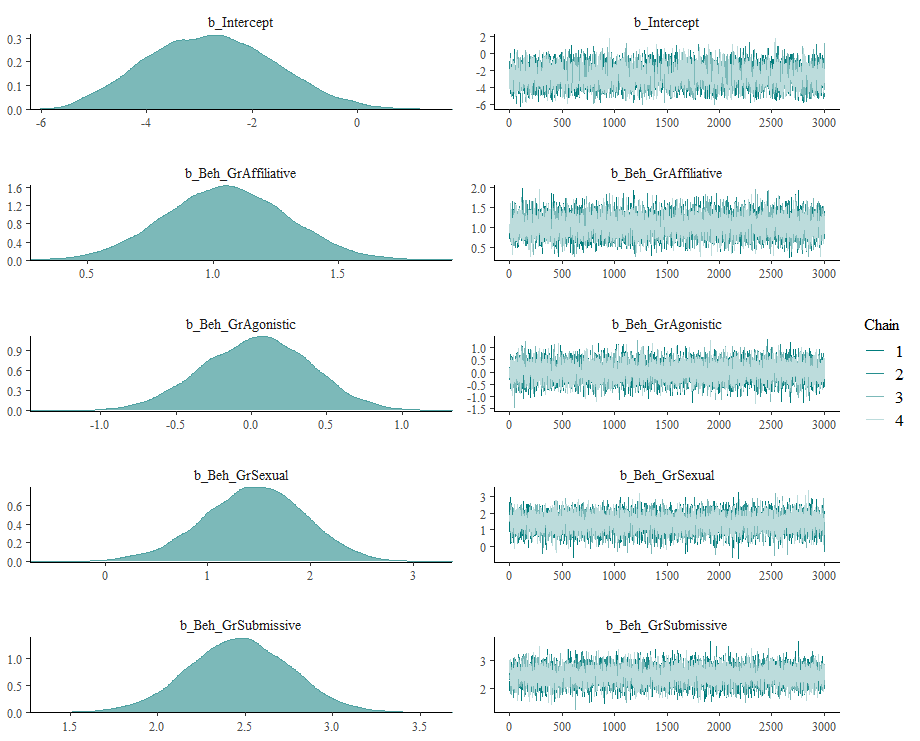
Model 1**

**
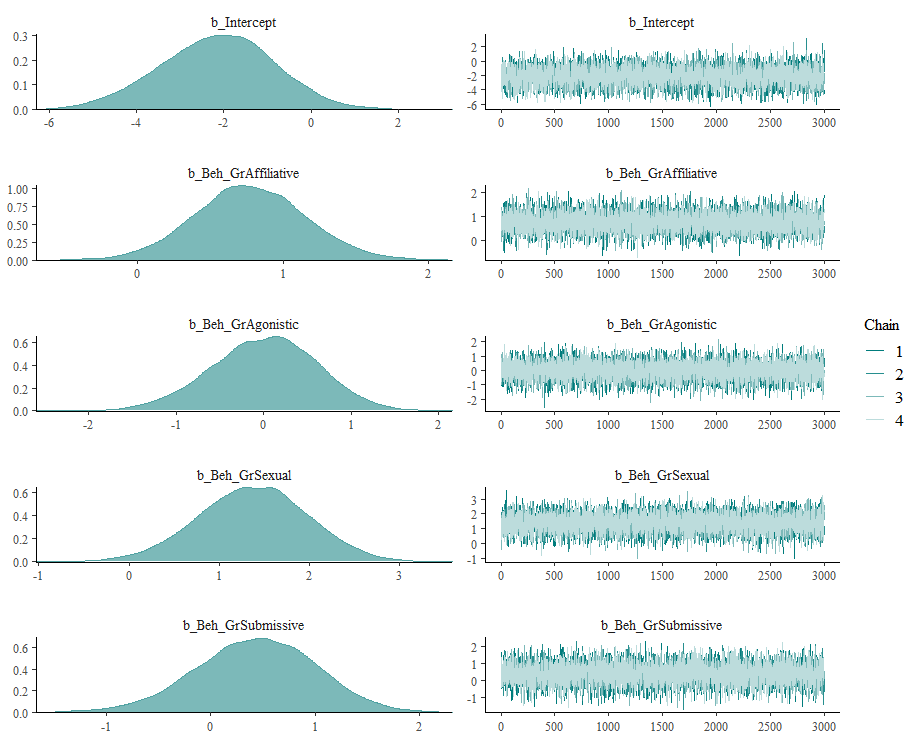

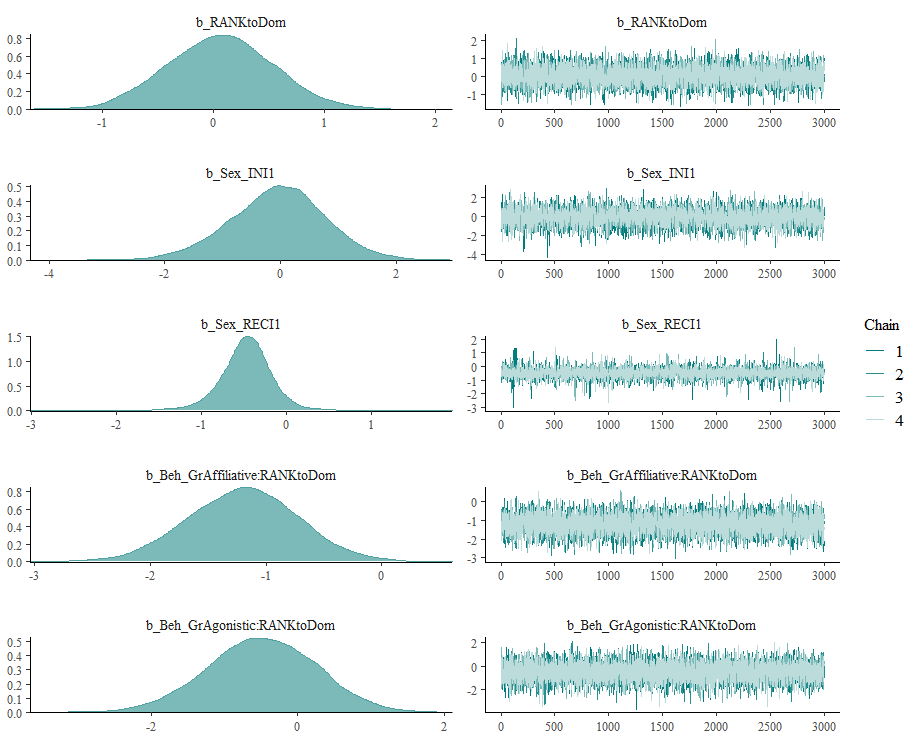

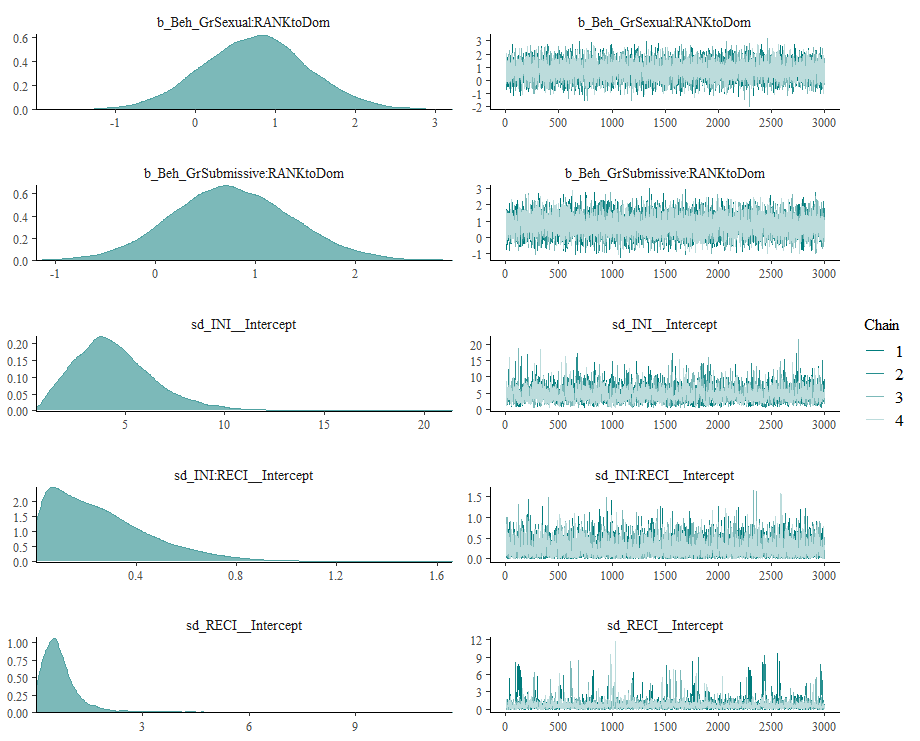
**
**Model 1a**

**Model 1b**

**
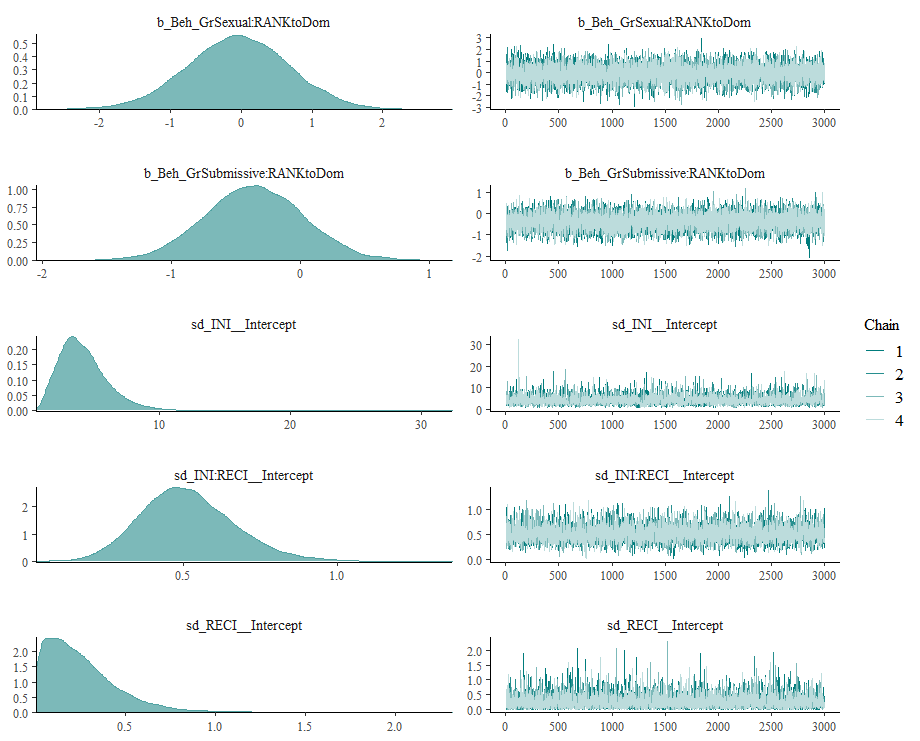

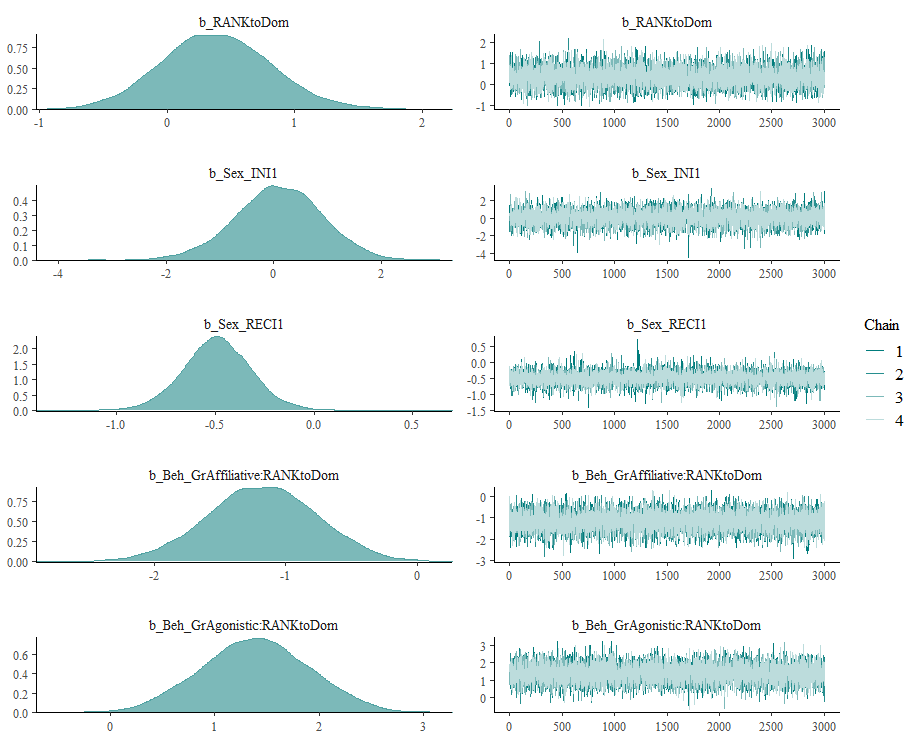

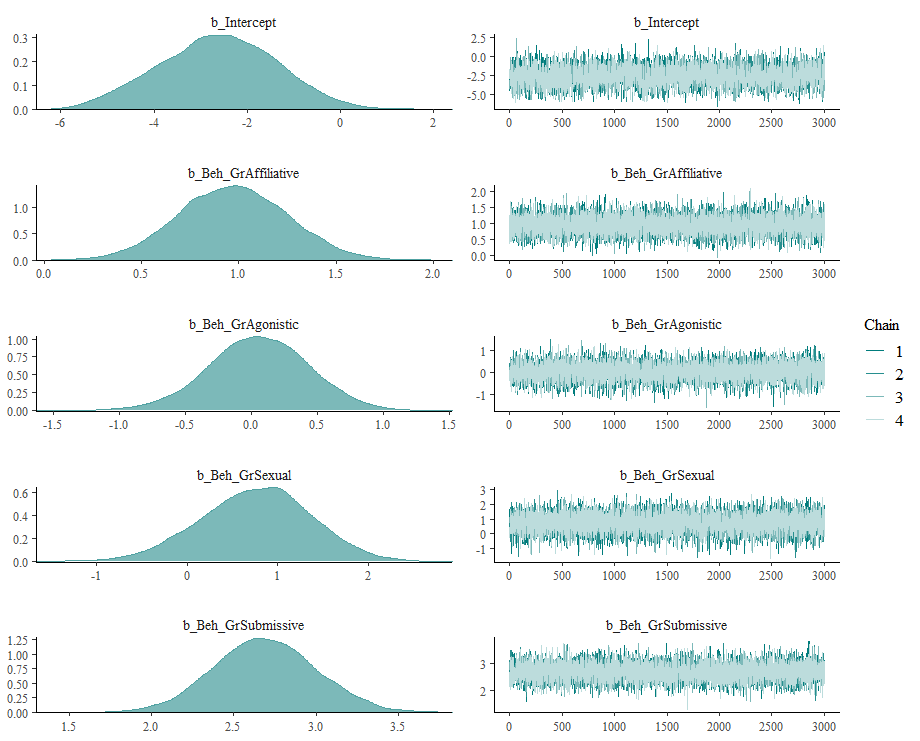
**

**Model 2**

*
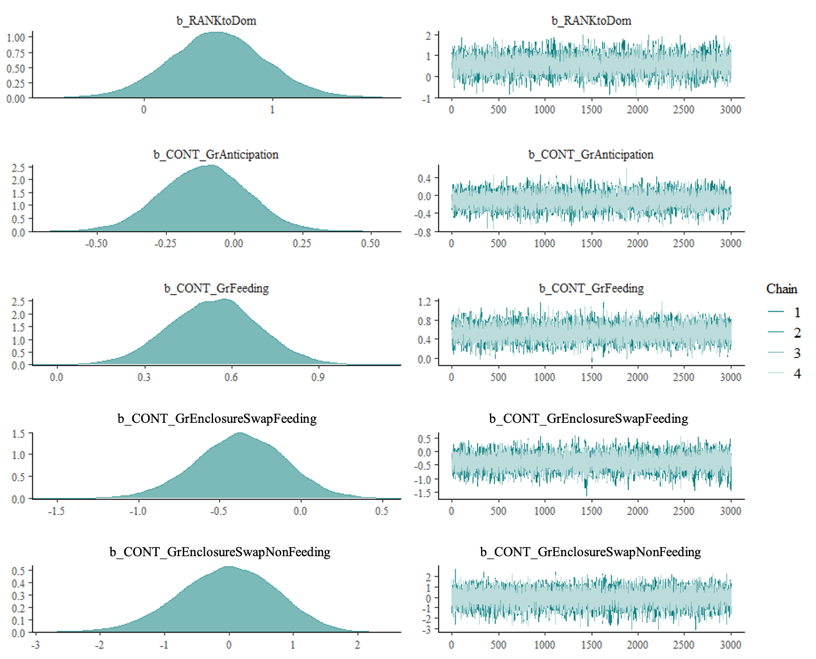
***
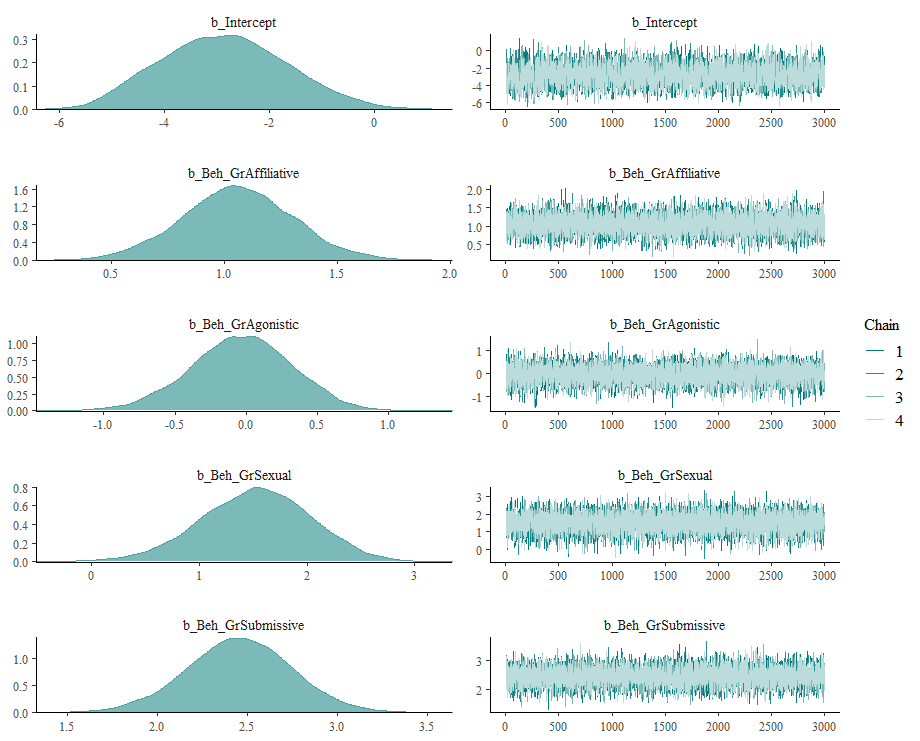
Model 2**

*
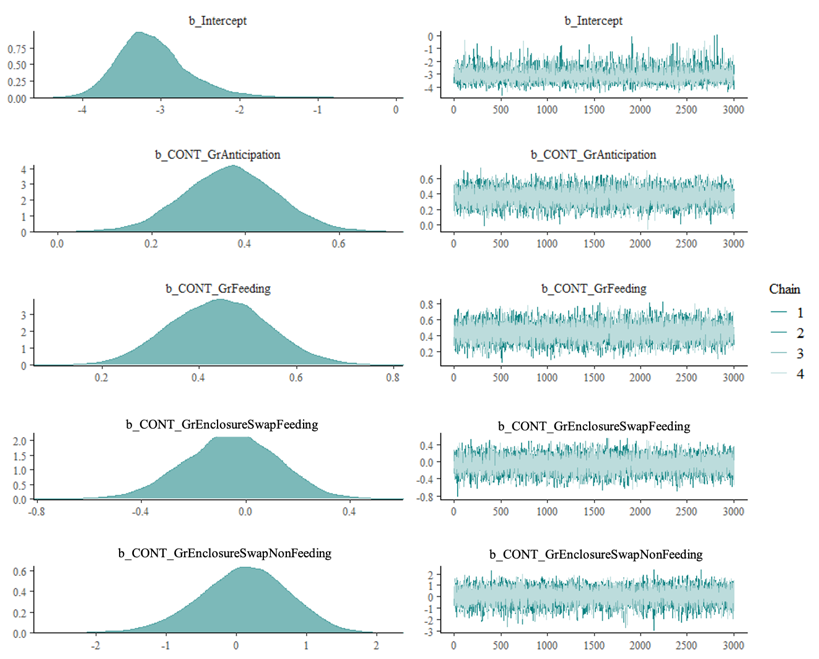
***
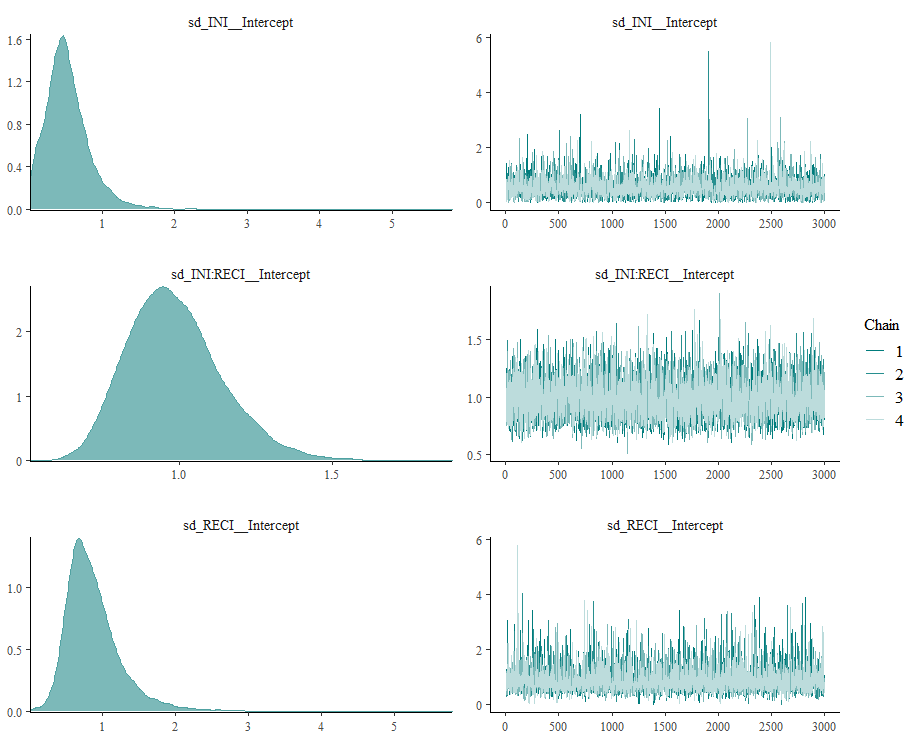
Model 2a**

*Figure S5.* MCMC trace-plots of posterior distributions of the Bayesian generalized linear mixed models. **Model 1:** Effects of rank and social contexts on the *bared-teeth display*. **Model 1a:** Effects of rank and social contexts on the *silent* *bared-teeth display*. **Model 1b:** effects of rank and social contexts on the *silent* *bared-teeth display*. **Model 2:** Effects of feeding conditions on the *bared-teeth display.* **Model 2a:** Effects of feeding conditions on aggressive behaviors.

**2. RESULTS**

Table S4. ***A.*** *Matrix of submissive behaviors upon aggression, including* Bend away, Flee, Avoid/Yielding, Retreat, Roll. ***B.*** *Matrix of aggressive behaviors, including* Aggression with attack, Charge/chase, Club, Direct display, Displacement, Retaliate, Shielding, Stealing/Take, Tease, Threat, Throw at. **C.** Dominance hierarchy results.

1. **Submissive upon aggression matrix**

|  | Ajani | Amber | Leen | Margo | Quincy | Saphira | Vizuri | Wakili |
| --- | --- | --- | --- | --- | --- | --- | --- | --- |
| Ajani | 0 | 11 | 16 | 41 | 8 | 12 | 50 | 265 |
| Amber | 17 | 0 | 8 | 2 | 2 | 12 | 9 | 40 |
| Leen | 8 | 1 | 0 | 3 | 1 | 4 | 5 | 79 |
| Margo | 6 | 2 | 2 | 0 | 0 | 6 | 0 | 4 |
| Quincy | 10 | 3 | 11 | 5 | 0 | 4 | 8 | 34 |
| Saphira | 24 | 4 | 5 | 5 | 5 | 0 | 13 | 121 |
| Vizuri | 6 | 7 | 6 | 2 | 3 | 4 | 0 | 69 |
| Wakili | 3 | 0 | 7 | 5 | 3 | 4 | 6 | 0 |

1. **Aggression matrix**

|  | Ajani | Amber | Leen | Margo | Quincy | Saphira | Vizuri | Wakili |
| --- | --- | --- | --- | --- | --- | --- | --- | --- |
| Ajani | 0 | 0 | 27 | 57 | 8 | 50 | 11 | 5 |
| Amber | 8 | 0 | 4 | 4 | 2 | 9 | 3 | 20 |
| Leen | 31 | 7 | 0 | 0 | 9 | 14 | 7 | 13 |
| Margo | 82 | 8 | 0 | 0 | 0 | 5 | 0 | 0 |
| Quincy | 14 | 0 | 1 | 0 | 0 | 5 | 1 | 3 |
| Saphira | 27 | 5 | 3 | 1 | 3 | 0 | 2 | 7 |
| Vizuri | 42 | 9 | 2 | 1 | 8 | 14 | 0 | 7 |
| Wakili | 130 | 10 | 33 | 7 | 12 | 49 | 21 | 0 |

1. **Results of the dominance analysis**

| **Chimpanzee individuals**  **(from high to low ranking)** | **Rank Order** | **Normalized David’s Scores** |
| --- | --- | --- |
| Wakili | 1 | 5.897 |
| Margo | 2 | 4.310 |
| Leen | 3 | 4.138 |
| Vizuri | 4 | 3.935 |
| Amber | 5 | 2.945 |
| Ajani | 6 | 2.525 |
| Saphira | 7 | 2.276 |
| Quincy | 8 | 1.974 |

Table S5. *Model output for the association between the SBT display and social contexts and rank dyadic rank relationships (Model 1a).*

| Parameter | Median Estimate | MAD | 89% CI  lower bound | 89% CI  upper bound | *PD* |
| --- | --- | --- | --- | --- | --- |
| Intercept | -2.11 | 1.31 | -3.74 | -0.546 | 0.95 |
| Rank (to Dominant) | 0.068 | 0.479 | -0.51 | 0.665 | 0.55 |
| **Social context (Affiliative)** | 0.766 | 0.376 | 0.32 | 1.25 | 0.98 |
| Social context (Aggressive) | 0.030 | 0.609 | -0.727 | 0.753 | 0.52 |
| **Social context (Sexual)** | 1.38 | 0.606 | 0.648 | 2.13 | 0.99 |
| Social context (Submissive) | 0.444 | 0.574 | -0.263 | 1.14 | 0.77 |
| Sex of the initiator (Female) | -0.027 | 0.792 | -1.04 | 0.954 | 0.51 |
| Sex of the recipient (Female) | -0.461 | 0.267 | -0.822 | -0.118 | 0.94 |
| **Social context (Affiliative): Rank (to Dominant)** | -1.19 | 0.476 | -1.77 | -0.61 | 0.99 |
| Social context (Aggressive): Rank (to Dominant) | -0.488 | 0.749 | -1.39 | 0.427 | 0.74 |
| Social context (Sexual): Rank (to Dominant) | 0.76 | 0.648 | -0.036 | 1.58 | 0.87 |
| Social context (Submissive): Rank (to Dominant) | 0.755 | 0.596 | 0.058 | 1.53 | 0.90 |
| Random effects |  |  |  |  |  |
| *SD* (Intercept) Initiator | 4.24 | 1.9 | 2.21 | 7.11 |  |
| *SD* (Intercept) Initiator: Recipient | 0.218 | 0.194 | 0.047 | 0.537 |  |
| *SD* (Intercept) Recipient | 0.588 | 0.397 | 0.196 | 1.34 |  |

**Note*. The parameters in bold indicate robust effects. *Explanation of the statistical terms:* Median estimate = the estimate mean of the posterior distribution; MAD = median absolute deviation; CI = Two-sided 89% Credible intervals based on quantiles; PD = proportion of the posterior distribution that is of the median’s sign. Strongly correlated to the frequentist p-value (0.1, 0.05, 0.01 and 0.001 respectively correspond approximately to a *pd* of 95%, 97.5%, 99.5% and 99.95%). From the *brms* package (Bürkner, 2017).

Table S6. *Model output for the association between the VBT display and social contexts and dyadic rank relationships (Model 1b).*

| Parameter | Median Estimate | MAD | 89% CI  lower bound | 89% CI  upper bound | *PD* |
| --- | --- | --- | --- | --- | --- |
| Intercept | -2.6 | 1.29 | -4.19 | -1.06 | 0.98 |
| Rank (to Dominant) | 0.378 | 0.428 | -0.125 | 0.924 | 0.81 |
| **Social context (Affiliative)** | 0.97 | 0.283 | 0.637 | 1.32 | 1.00 |
| Social context (Aggressive) | 0.0531 | 0.381 | -0.404 | 0.529 | 0.55 |
| Social context (Sexual) | 0.779 | 0.61 | 0.006 | 1.52 | 0.88 |
| **Social context (Submissive)** | 2.69 | 0.312 | 2.32 | 3.09 | 1.00 |
| Sex of the initiator (Female) | 0.1 | 0.801 | -0.879 | 1.09 | 0.55 |
| **Sex of the recipient (Female)** | -0.492 | 0.171 | -0.701 | -0.275 | 0.99 |
| **Social context (Affiliative): Rank (to Dominant)** | -1.18 | 0.419 | -1.68 | -0.653 | 1.00 |
| **Social context (Aggressive): Rank (to Dominant)** | 1.39 | 0.52 | 0.761 | 2.05 | 1.00 |
| Social context (Sexual): Rank (to Dominant) | -0.039 | 0.713 | -0.91 | 0.848 | 0.52 |
| Social context (Submissive): Rank (to Dominant) | -0.355 | 0.378 | -0.809 | 0.121 | 0.82 |
| Random effects |  |  |  |  |  |
| *SD* (Intercept) Initiator | 3.96 | 1.7 | 2.23 | 6.68 |  |
| *SD* (Intercept) Initiator: Recipient | 0.502 | 0.149 | 0.336 | 0.71 |  |
| *SD* (Intercept) Recipient | 0.21 | 0.18 | 0.045 | 0.515 |  |

**Note*. The parameters in bold indicate robust effects. *Explanation of the statistical terms:* MAD = median absolute deviation; CI = Two-sided 89% Credible intervals based on quantiles; PD = proportion of the posterior distribution that is of the median’s sign. Strongly correlated to the frequentist p-value (0.1, 0.05, 0.01 and 0.001 respectively correspond approximately to a *pd* of 95%, 97.5%, 99.5% and 99.95%). From the *brms* package (Bürkner, 2017).

Table S7. *Model output for the association between the BT display and environmental conditions (Model 2).*

| Parameter | Median Estimate | MAD | 89% CI  lower bound | 89% CI  upper bound | *PD* |
| --- | --- | --- | --- | --- | --- |
| Intercept | -2.88 | 1.25 | -4.35 | -1.31 | 0.99 |
| Rank (to Dominant) | 0.571 | 0.365 | 0.126 | 1.04 | 0.93 |
| **Social context (Affiliative)** | 1.06 | 0.24 | 0.765 | 1.36 | 1.00 |
| Social context (Aggressive) | -0.03 | 0.356 | -0.466 | 0.416 | 0.53 |
| **Social context (Sexual)** | 1.53 | 0.51 | 0.91 | 2.16 | 1.00 |
| **Social context (Submissive)** | 2.46 | 0.288 | 2.11 | 2.81 | 1.00 |
| Sex of the initiator (Female) | 0.165 | 0.758 | -0.805 | 1.1 | 0.58 |
| **Sex of the recipient (Female)** | -0.502 | 0.15 | -0.687 | -0.312 | 1.00 |
| **Social context (Affiliative): Rank (to Dominant)** | -1.4 | 0.338 | -1.82 | -0.987 | 1.00 |
| Social context (Aggressive): Rank (to Dominant) | 0.909 | 0.482 | 0.337 | 1.51 | 0.97 |
| Social context (Sexual): Rank (to Dominant) | 0.421 | 0.583 | -0.274 | 1.16 | 0.77 |
| Social context (Submissive): Rank (to Dominant) | -0.408 | 0.336 | -0.805 | 0.021 | 0.88 |
| Environmental condition (Anticipation) | -0.108 | 0.157 | -0.297 | 0.085 | 0.75 |
| **Environmental condition (Feeding)** | 0.542 | 0.154 | 0.351 | 0.733 | 1.00 |
| Environmental condition (EnclosureSwapFeeding) | -0.37 | 0.273 | -0.704 | -0.037 | 0.91 |
| Environmental condition (EnclosureSwapNonFeeding) | -0.01 | 0.759 | -0.947 | 0.9 | 0.50 |
| Random effects |  |  |  |  |  |
| *SD* (Intercept) Initiator | 3.5 | 1.55 | 1.95 | 5.94 |  |
| *SD* (Intercept) Initiator: Recipient | 0.54 | 0.132 | 0.399 | 0.728 |  |
| *SD* (Intercept) Recipient | 0.149 | 0.136 | 0.0316 | 0.403 |  |

**Note*. The parameters in bold indicate robust effects. *Explanation of the statistical terms:* MAD = median absolute deviation; CI = Two-sided 89% Credible intervals based on quantiles; PD = proportion of the posterior distribution that is of the median’s sign. Strongly correlated to the frequentist p-value (0.1, 0.05, 0.01 and 0.001 respectively correspond approximately to a *pd* of 95%, 97.5%, 99.5% and 99.95%). From the *brms* package (Bürkner, 2017).

Table S8. *Model output for the association between the environmental conditions and aggression (Model 2a).*

| Parameter | Median Estimate | MAD | 89% CI  lower bound | 89% CI  upper bound | *PD* |
| --- | --- | --- | --- | --- | --- |
| Intercept | -3.15 | 0.411 | -3.6 | -2.51 | 1.00 |
| **Environmental condition (Anticipation)** | 0.368 | 0.098 | 0.25 | 0.491 | 1.00 |
| **Environmental condition (Feeding)** | 0.442 | 0.102 | 0.319 | 0.569 | 1.00 |
| Environmental condition (EnclosureSwapFeeding) | -0.051 | 0.177 | -0.273 | 0.166 | 0.61 |
| Environmental condition (EnclosureSwapNonFeeding) | 0.128 | 0.628 | -0.666 | 0.893 | 0.58 |
| Random effects |  |  |  |  |  |
| *SD* (Intercept) Initiator | 0.48 | 0.256 | 0.194 | 0.889 |  |
| *SD* (Intercept) Initiator: Recipient | 0.97 | 0.149 | 0.808 | 1.19 |  |
| *SD* (Intercept) Recipient | 0.804 | 0.308 | 0.5 | 1.36 |  |

**Note*. The parameters in bold indicate robust effects. *Explanation of the statistical terms:* MAD = median absolute deviation; CI = Two-sided 89% Credible intervals based on quantiles; PD = proportion of the posterior distribution that is of the median’s sign. Strongly correlated to the frequentist p-value (0.1, 0.05, 0.01 and 0.001 respectively correspond approximately to a *pd* of 95%, 97.5%, 99.5% and 99.95%). From the *brms* package (Bürkner, 2017).

**References:**

Bürkner, P.-C. (2017). Advanced Bayesian multilevel modeling with the R package brms. *arXiv preprint arXiv:1705.11123*.

Carlsen, F., & de Jongh, T. (2007). European studbook for the chimpanzee Pan troglodytes. *Copenhagen Zoo. Roskildevej*, *38*, 1-278.

Cronin, K. A., De Groot, E., & Stevens, J. M. (2015). Bonobos show limited social tolerance in a group setting: A comparison with chimpanzees and a test of the relational model. *Folia primatologica*, *86*(3), 164-177.

de Waal, F. B. (1988). The communicative repertoire of captive bonobos (*Pan paniscus*), compared to that of chimpanzees. *Behaviour*, *106*(3-4), 183-251.

Friard, O., & Gamba, M. (2016). BORIS: a free, versatile open‐source event‐logging software for video/audio coding and live observations. *Methods in Ecology and Evolution*, *7*(11), 1325-1330.

Goodall, J. (1986). The chimpanzees of Gombe: Patterns of behavior. *Cambridge Mass*.

Hobaiter, C., & Byrne, R. W. (2011). Serial gesturing by wild chimpanzees: its nature and function for communication. *Animal cognition*, *14*(6), 827-838.

Nishida, T., Kano, T., Goodall, J., McGrew, W. C., & Nakamura, M. (1999). Ethogram and ethnography of Mahale chimpanzees. *Anthropological Science*, *107*(2), 141-188.

Palagi, E. (2008). Sharing the motivation to play: the use of signals in adult bonobos. *Animal behaviour*, *75*(3), 887-896.

Parr, L. A., Cohen, M., & De Waal, F. (2005). Influence of social context on the use of blended and graded facial displays in chimpanzees. *International journal of primatology*, *26*(1), 73-103.

Pollick, A. S., & De Waal, F. B. (2007). Ape gestures and language evolution. *Proceedings of the National Academy of Sciences*, *104*(19), 8184-8189.

Van Hooff, J. (1973). A structure analysis of the social behaviour of a semi-captive group of chimpanzees. *Social communication and movement*, 75-162.

Vlaeyen, J., Heesen, R., Clay, Z., Kret, M. E., & Kim, Y. (in revision). Bared-teeth display in bonobos (*Pan paniscus*).
